# Inter-Country COVID-19 Contagiousness Variation in Eight African Countries

**DOI:** 10.3389/fpubh.2022.796501

**Published:** 2022-06-02

**Authors:** Geoffrey Chiyuzga Singini, Samuel O. M. Manda

**Affiliations:** ^1^Chancellor College, University of Malawi, Zomba, Malawi; ^2^Biostatistics Research Unit, South Africa Medical Research Council, Pretoria, South Africa; ^3^Department of Statistics, University of Pretoria, Pretoria, South Africa

**Keywords:** COVID-19, reproduction number, contact rate, removal rate, infectious period, Africa

## Abstract

The estimates of contiguousness parameters of an epidemic have been used for health-related policy and control measures such as non-pharmaceutical control interventions (NPIs). The estimates have varied by demographics, epidemic phase, and geographical region. Our aim was to estimate four contagiousness parameters: basic reproduction number (*R*_0_), contact rate, removal rate, and infectious period of coronavirus disease 2019 (COVID-19) among eight African countries, namely Angola, Botswana, Egypt, Ethiopia, Malawi, Nigeria, South Africa, and Tunisia using Susceptible, Infectious, or Recovered (SIR) epidemic models for the period 1 January 2020 to 31 December 2021. For reference, we also estimated these parameters for three of COVID-19's most severely affected countries: Brazil, India, and the USA. The basic reproduction number, contact and remove rates, and infectious period ranged from 1.11 to 1.59, 0.53 to 1.0, 0.39 to 0.81; and 1.23 to 2.59 for the eight African countries. For the USA, Brazil, and India these were 1.94, 0.66, 0.34, and 2.94; 1.62, 0.62, 0.38, and 2.62, and 1.55, 0.61, 0.39, and 2.55, respectively. The average COVID-19 related case fatality rate for 8 African countries in this study was estimated to be 2.86%. Contact and removal rates among an affected African population were positively and significantly associated with COVID-19 related deaths (*p*-value < 0.003). The larger than one estimates of the basic reproductive number in the studies of African countries indicate that COVID-19 was still being transmitted exponentially by the 31 December 2021, though at different rates. The spread was even higher for the three countries with substantial COVID-19 outbreaks. The lower removal rates in the USA, Brazil, and India could be indicative of lower death rates (a proxy for good health systems). Our findings of variation in the estimate of COVID-19 contagiousness parameters imply that countries in the region may implement differential COVID-19 containment measures.

## 1. Introduction

The coronavirus disease 2019 (COVID-19) pandemic, which originated in Wuhan in China in December (1, 2), has now infected over 304 million people and caused more than 5.4 million deaths (3). As of 26 December 2021, the African continent had 7,055,628 confirmed cases and 1,55,292 cumulative deaths, while the global total of 278.7 million COVID-19 infected people with over 5.39 million deaths had been reported by WHO (4). Due to the slow roll-out of COVID-19 vaccinations, especially in the African continent (3), non-pharmaceutical control measures such as social distancing, travel and border closures, school closures, isolation of symptomatic individuals and their contacts, and large-scale lockdowns of populations have been by far the main containment measures against the pandemic spread.

Coronavirus disease 2019 non-pharmaceutical policy responses have largely depended on epidemiological parameters of the pandemic estimated from mathematical and statistical COVID-19 modeling.The models have included epidemiological growth models (5–8), and the Susceptible, Infected, and Recovered (SIR) type models (9–15). In other epidemiological, healthcare, and surveillance indicators, the estimates have provided relevant policymakers with scientifically driven strategies for appropriately imposing and lifting COVID-19 related restrictions. Our study used the Susceptible, Infectious, or Recovered (*SIR*) modeling approach as it does not depend on the data, uses fewer assumptions, and has good predictive power (16, 17).

However, a classic SIR model's estimation of COVID-19 contagiousness parameters, namely the effective reproductive number, contact rate, removal rate, and infectious period, assumes a homogeneous mixing of the infected and susceptible populations. The total population is constant in time. This may not be the case when several communities or countries are analyzed since these may differ in COVID-19 venerability risk factors, disease burden, health systems, and changes in testing policies resulting in variations in infections detected over time and between countries (18–20). As regards countries in Africa, there is a paucity of studies that have looked at differences in contagiousness parameters of COVID-19 infections based on fitting SIR-type mathematical models. Countries in Africa have great variation in socioeconomic and COVID-19 health vulnerabilities (20, 21). Thus, it is reasonable to assume that there would be differences in COVID-19 contagiousness parameters from fitting SIR models. This article analyses COVID-19 data from eight purposely selected African countries, namely Angola, Botswana, Egypt, Ethiopia, Malawi, Nigeria, South Africa, and Tunisia, using SIR models. The country-specific estimates of the COVID-19 contagiousness parameters were compared to those obtained from an analysis of the three hardest-hit countries, Brazil, India, and the USA.

## 2. Methodology and Data Source

### Settings

We studied eight African countries; Angola, Botswana, Egypt, Ethiopia, Malawi, Nigeria, South Africa, and Tunisia, which were chosen subjectively. For comparison, the three hardest-hit countries, Brazil, India, and the USA were also included in our analysis. Table 1 shows several COVID-19 vulnerability risk factors, including total populations, GDPs, the proportion of the elderly populations, international exposure, and population density of the selected countries. There is so much difference in the African countries concerning all risk factors, e.g., GDPs (with Botswana and South Africa being the wealthiest) and population density (with Botswana having the lowest).

**Table 1:** Summary of the country context.

	**USA**	**Brazil**	**India**	**Angola**	**Botswana**	**Egypt**	**Ethiopia**	**Malawi**	**Nigeria**	**South Africa**	**Tunisia**
Total Population	331,002,651	214,619,177	1,380,004,385	32,866,272	2,351,627	102,334,404	114,963,588	19,129,952	206,139,589	59,308,690	11,818,619
Percentage of Female	50.52%	50.87%	48.04%	50.52%	51.60%	49.50%	50.00%	50.68%	49.30%	50.70%	50.40%
GDP per capita	54225.45	14103.45	6426.67	5819.50	15807.37	10550.21	1729.93	1095.04	5338.45	12294.88	10849.30
Proportion Aged 65 yrs	15.41	8.55	5.99	2.41%	3.94	5.16	3.53	2.98	2.75	5.34	8.00
COVID19 Total Tested	583.91million	57.5million	569.0million		1.8million		3.5million	309766	3.0million	17.7million	2.9million
Daily COVID-19 case fatality rate	1.61%	2.79%	1.60%	2.72%	1.32%	5.69%	1.61%	3.71%	1.32%	3.02%	3.52%
Density (P/sq. KM)	36	25	464	26	4	103	115	203	226	49	76
Fertility Rate	1.78	1.70	2.24	5.55	2.89	3.33	4.30	4.25	5.42	2.41	2.20
Median Age	38	34	28	17	24	25	19	18	18	28	33
Total Deaths due to COVID-19	701201	596749	448339	1746	2427	21752	6898	2332	3022	90814	25569
Proportion of people who received ≥ 1 dose	63.60%	69.90%	49.60%	13.10%	45.90%	24.30%	1.40%	4.00%	2.50%	27.60%	52.40%
Prevalence of Diabetes	10.79	8.11	10.39	3.94%	4.81	17.31	7.47	3.94	2.42	5.52	8.52
Mortality rate due to cardiovascular	151.09	177.96	282.28	276.05	237.37	525.43	182.63	227.35	181.01	200.38	318.99
International Exposure				3	4	5	3	2	5	5	4
Public Health Systems				4	2	1	3	3	5	2	1
Density of urban areas				4	1	3	4	1	2	1	2
Total Populations in Urban Areas				3	1	5	5	2	5	4	2
Government Transparency				4	1	3	2	3	4	1	1
Press Freedom				4	3	4	4	3	3	2	3
Conflict Magnitude				1	1	2	3	1	4	1	1
Forced Displacement				3	1	4	5	3	5	4	2

### 2.1. Data

Country-level cumulative COVID-19 cases and deaths were extracted for the period of 1 January 2020 to 31 December, 2021 from publicly available COVID-19 data at the Johns Hopkins Coronavirus Resource Center at: https://coronavirus.jhu.edu/about/how-to-use-our-data.

### 2.2. The SIR Model

Understanding dynamics and spread of an epidemic often relies on predictive mathematical epidemic models. These models consider the movement of of individual through mostly four mutually exclusive stages of infection: susceptible (S), exposed (E), infectious (I) and removed (R), giving rise to the SEIR model, which is a slight extension of the usual SIR model. Individuals vulnerable to infection belong to the S (susceptible) compartment. Those already infected but do not show symptoms or cannot infect others belong to the E (exposed) compartment. An infected individual who starts infecting others belongs to the I (infectious) compartment while those cured of the infection belong to the R (recovered) compartment. A recovered individual either remains there if they get permanent recovery or may become susceptible again and move back into the S compartment (10, 14, 22).

Many dynamic models for infectious diseases, such as *SIS*, *SIR*, *SEIS*, *SIS*, and *SIRS*, demonstrate that incidence increases with the numbers of susceptible, infectious, and saturation are incorporated into their mathematical forms for a better understanding of the epidemics (10). In these models, the population is assumed to be homogeneously mixed, and individuals get infections or are cured at constant rates. The Basic reproduction number (*R*_0_), a fundamental determinant of the dynamics of disease infection at the population level, offers insights into controlling the epidemic. When *R*_0_ > 1 an epidemic results in an outbreak. This threshold property provides important information about the potential of disease spread and the impact of control mechanisms. Our choice of the *SIR* model is motivated by its non-dependence on data, use of fewer assumptions, and the predictive power to show how different public health interventions affect the outcome of infectious diseases such as understanding patients' immunity in the absence of enough evidence (10, 11).


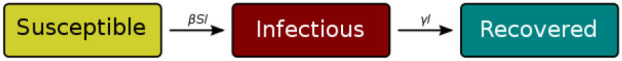



$$\frac{\text{d}S}{\text{d}t}=-\beta IS$$

$$\frac{\text{d}I}{\text{d}t}=\beta IS-\gamma I$$

$$\frac{\text{d}R}{\text{d}t}=\gamma I$$

where *S*(0) = *S*_0_ > 0, *I*(0) = *I*_0_ > 0, and *R*(*t*) = *R*_0_ = 0. Additionally, also


$$\frac{\text{d}S}{\text{d}t}+\frac{\text{d}I}{\text{d}t}+\frac{\text{d}R}{\text{d}t}=0$$

implying that this gives a constant term


S(t)+I(t)+R(t)=N

such that *S, I, R* are bounded by N. The dynamics of the infectious class depends on the following ratio:


$$R_{0}=\frac{\beta }{\gamma }$$

which is referred to as the basic reproduction ratio. The biological interpretation of the parameters is given in the table below:

**Table:** 

**Parameter**	**Biological meaning**
β	Contact rate
γ^−1^	Mean recovery rate for the clinically ill
S	Proportion of susceptible population
I	Proportion of infected population
R	Proportion of recovered population
N	Total population
S_0	Number of susceptible population at time *t* = 0
I_0	Number of infected population at time *t* = 0
R_0	Number of recovered population at time *t* = 0
S_c	Relative removal rate

### 2.3. Estimation of Model Parameters

The four contagiousness COVID-19 parameters in the *SIR* model described in Section 2.2 for each of the eight African countries and the three COVID-19 hardest-hit countries were estimated using the COVID-19 analytics R package which allows users to access and analyze worldwide data from resources publicly available (23). The package is easily accessible from https://github.com/mponce0/covid19.analytics. Even though there are many R packages and resources for analyzing the COVID-19 pandemic, the COVID-19 analytics package offers more analysis options, including estimates of growth rates and daily changes and dashboards. It is also easy to implement when estimating key epidemiological parameters of COVID-19 using SIR models.

Key epidemic indicators were calculated like the herd immunity threshold (HIT), which represents the minimum proportion of a population that must be immune by vaccination or natural infection to halt the unfolding of associated infection in a given community, defined as $HIT=1-\frac{1}{R_{0}}$ by Kwok et al. (24). We also used the basic reproduction number *R*_0_, defined as the average number of secondary infections when one infected individual is introduced into a completely susceptible population. We then estimated the contact rate (β) and removal rate (γ). From the removal rate, we determined the average infectious period to be ($\frac{1}{\gamma }$). Considering a homogeneous population, we interpret herd immunity to be achieved when $1-\frac{1}{R_{0}}$ of the population has become immune, either through the disease itself or vaccination (9, 25).

Once the parameters were estimated, we statistically performed the correlation analysis of basic reproduction number, removal rates, and infectivity period against some identified risk factors for COVID-19 to establish an association among the random variables from a univariate distribution perspective. The correlation study generated *p*-values which we used to make a determination of dependence between random variables.

## 3. Results

### Comparative Analysis

Previous studies have found estimates of *R*_0_ to range from 1.35 to 2.11, 1.5 to 2.0, and 1.41 to 2.12 for the USA, Brazil, and India, respectively (26–29). For our study, these were estimated at *R*_0_ = 1.94, *R*_0_ = 1.55, and *R*_0_ = 1.62 for USA, Brazil, and India, respectively.

Figures 1–9 present COVID-19 trajectories for each of the 8 African countries using the total number of confirmed COVID-19 cases and the global totals.

**Figure 1:**
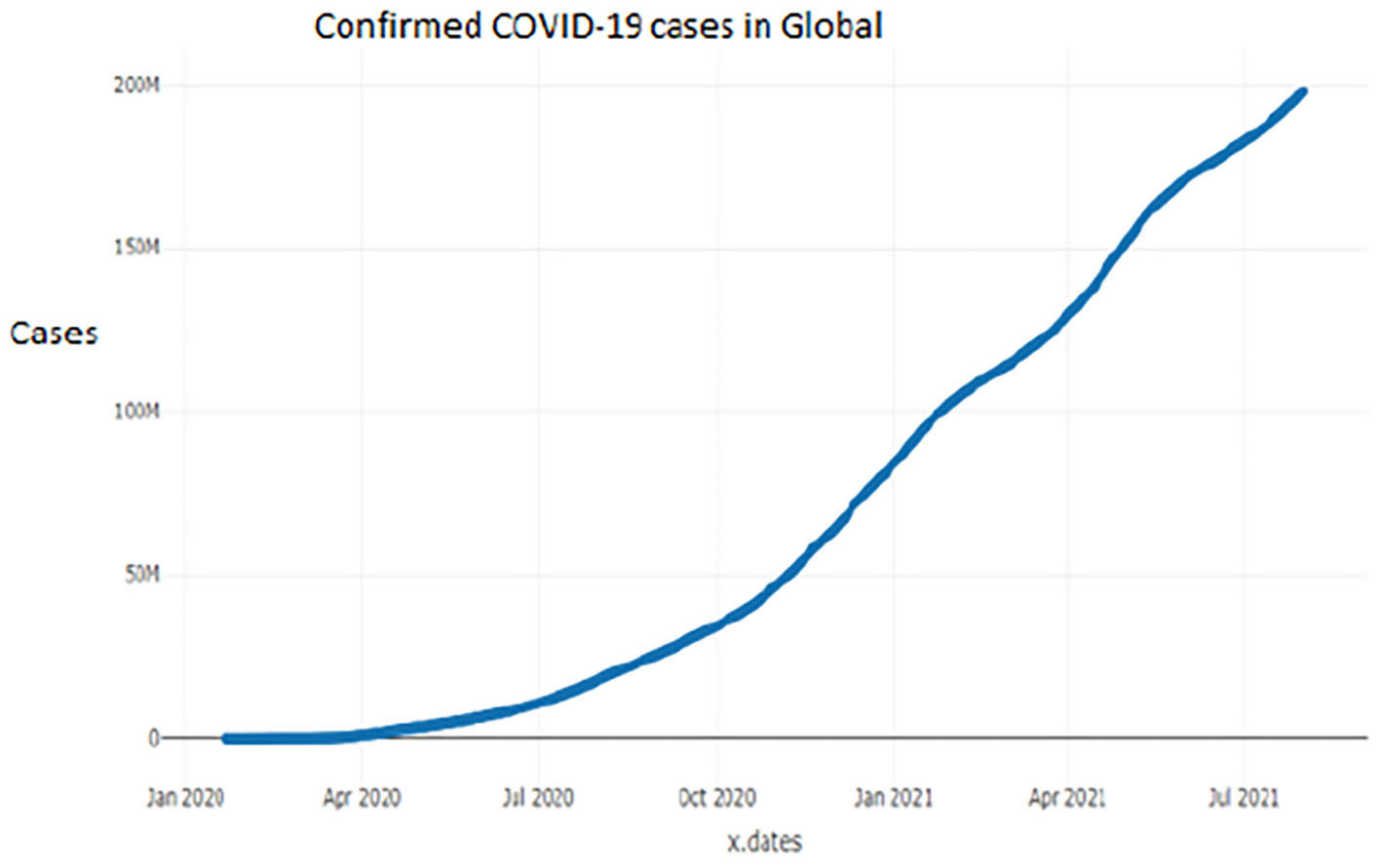
Global cases.

**Figure 2:**
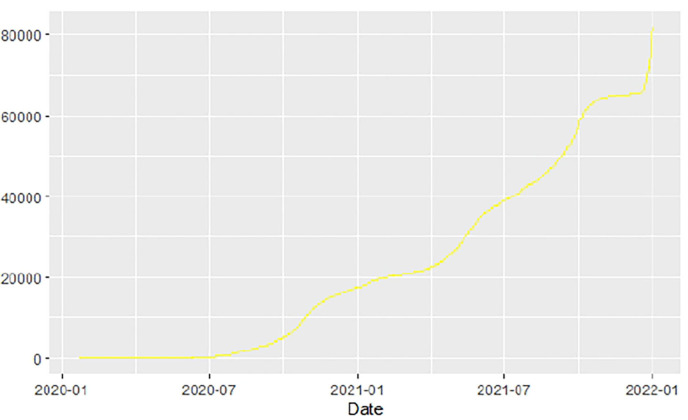
Angola cases.

**Figure 3:**
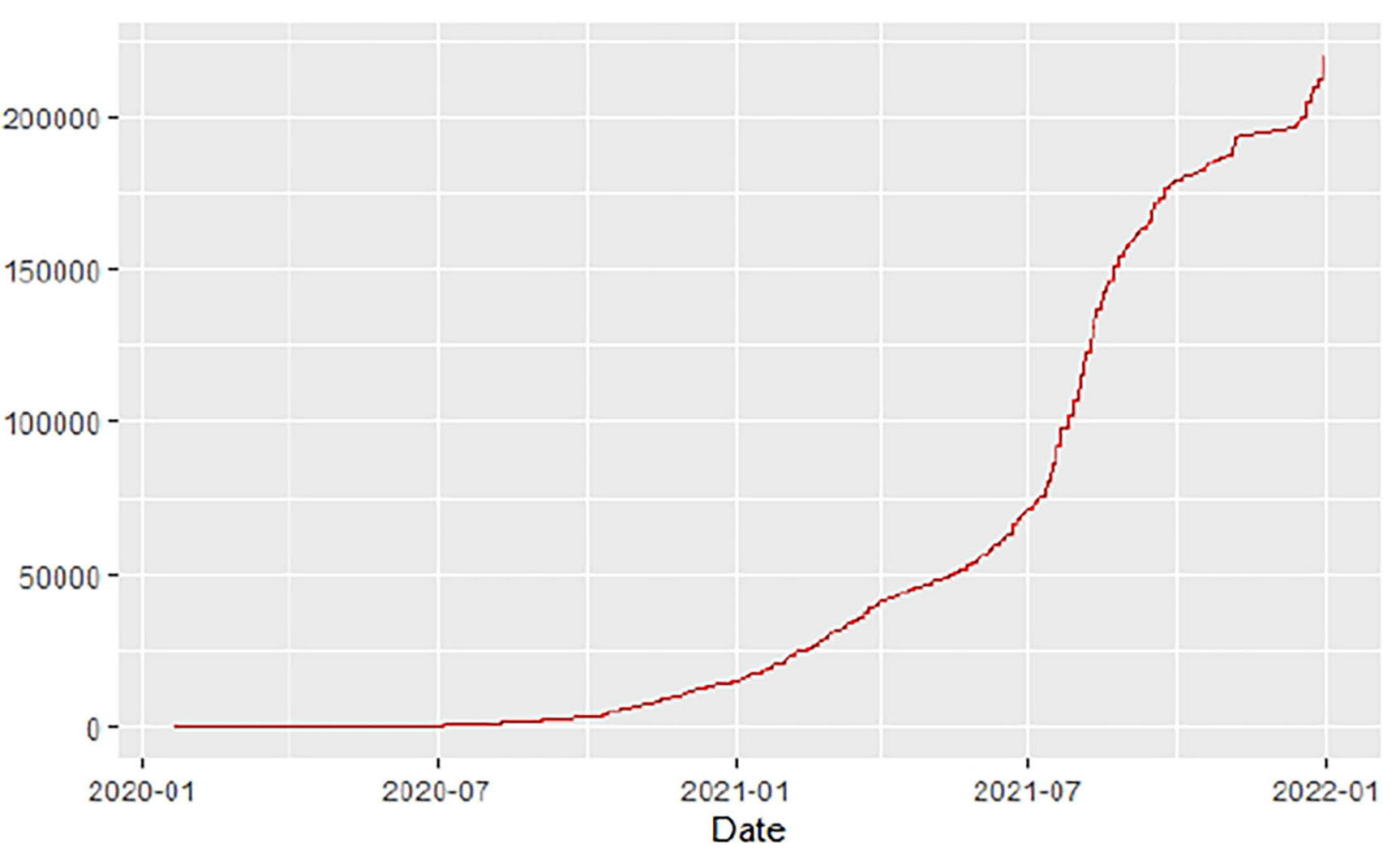
Botswana cases.

**Figure 4:**
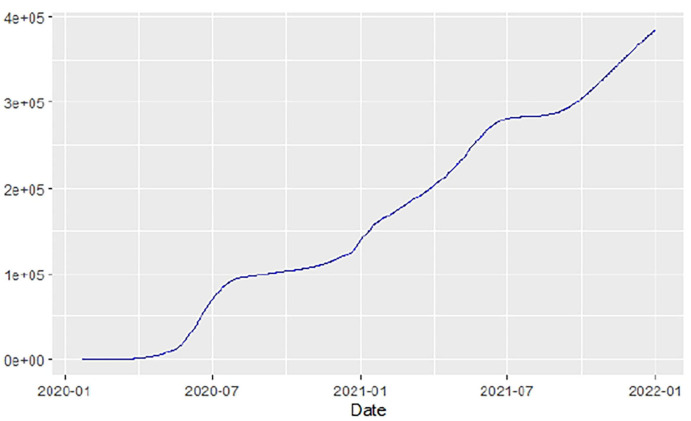
Egypt cases.

**Figure 5:**
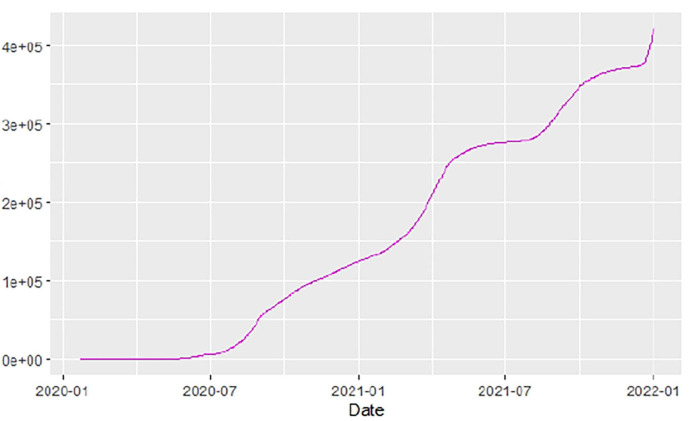
Ethiopia cases.

**Figure 6:**
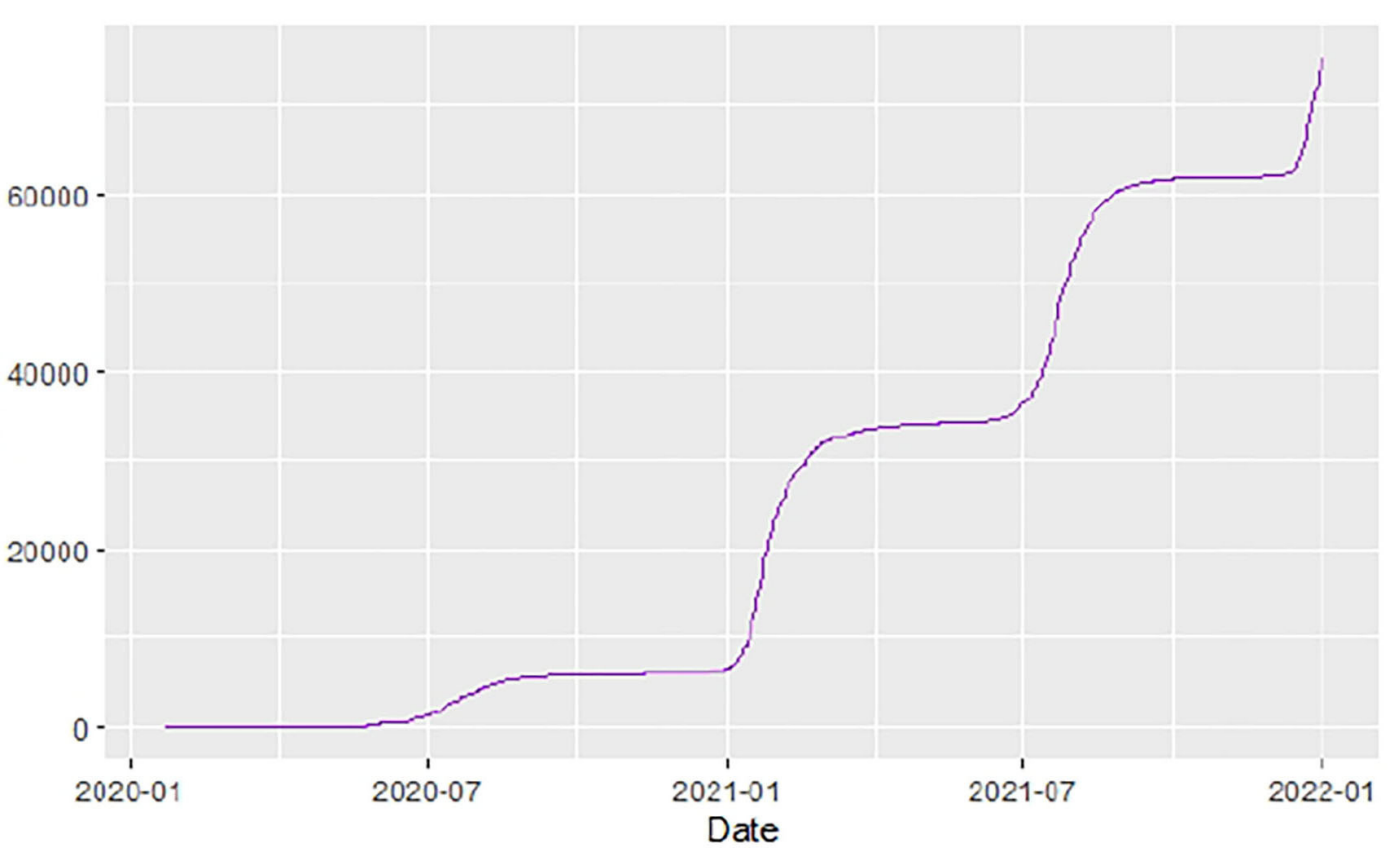
Malawi cases.

**Figure 7:**
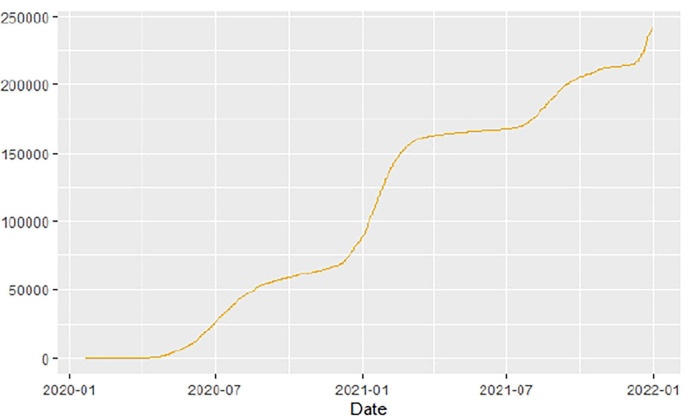
Nigeria cases.

**Figure 8:**
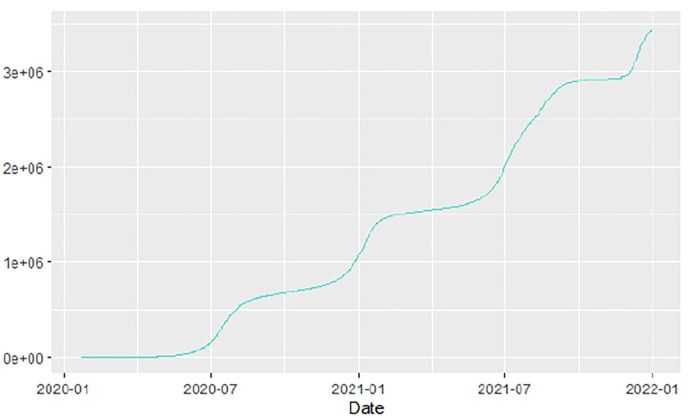
South Africa cases.

**Figure 9:**
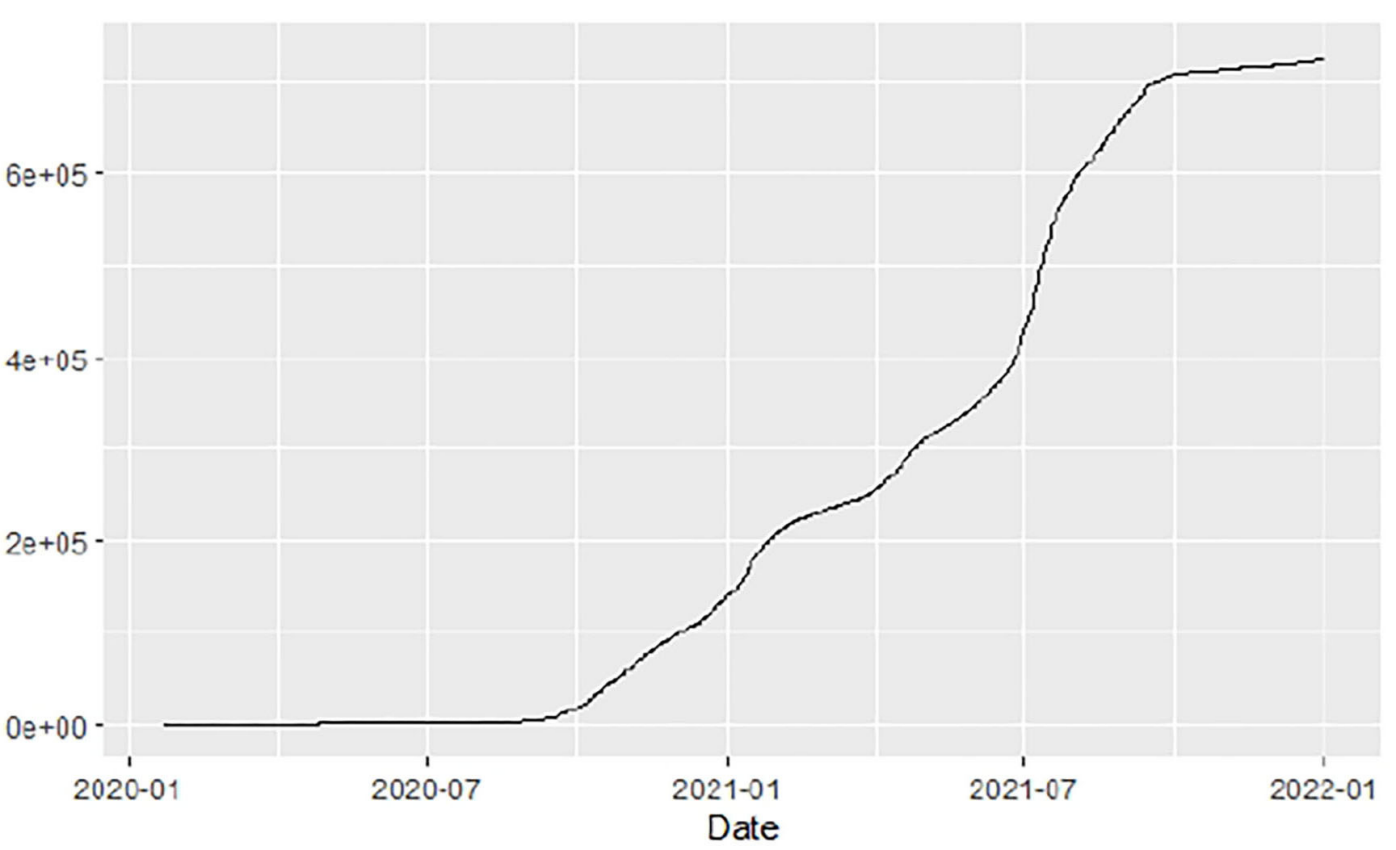
Tunisia cases.

The patterns display a general trend of exponential growth, and they are characterized by fluctuations within short time intervals showing rapid changes in cases confirmed. While the graphs confirmed that all the 8 countries experienced the two COVID-19 waves, Botswana and Tunisia show a steep growth in confirmed cases (refer to Figures 3, 9). For Botswana, the peaks for wave one and wave two were not easy to distinguish but observably close to each other.

Similarly, Figures 10–18 show COVID-19 death trajectories within the sampled countries compared to the total global deaths. The growth in fatalities is not very steep, suggesting it could be closer to a constant change in deaths. All the selected countries displayed similar patterns of deaths due to COVID-19 and an exponential growth pattern.

**Figure 10:**
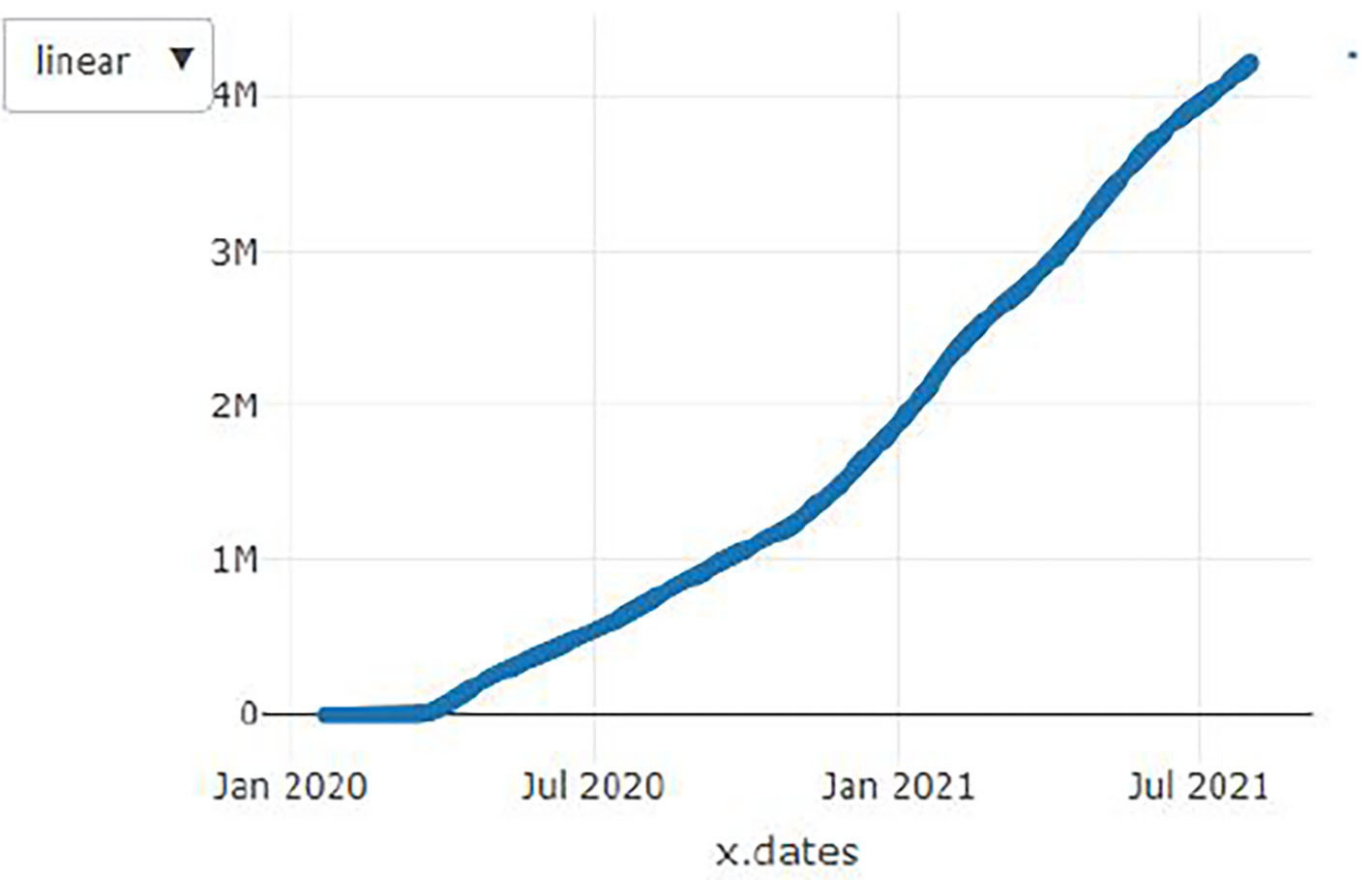
Global deaths.

**Figure 11:**
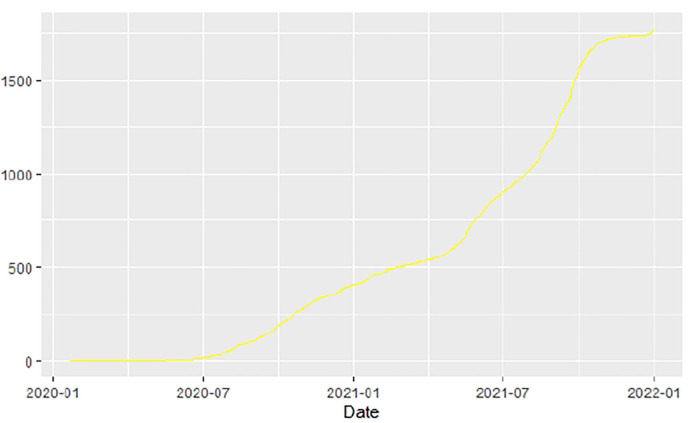
Angola deaths.

**Figure 12:**
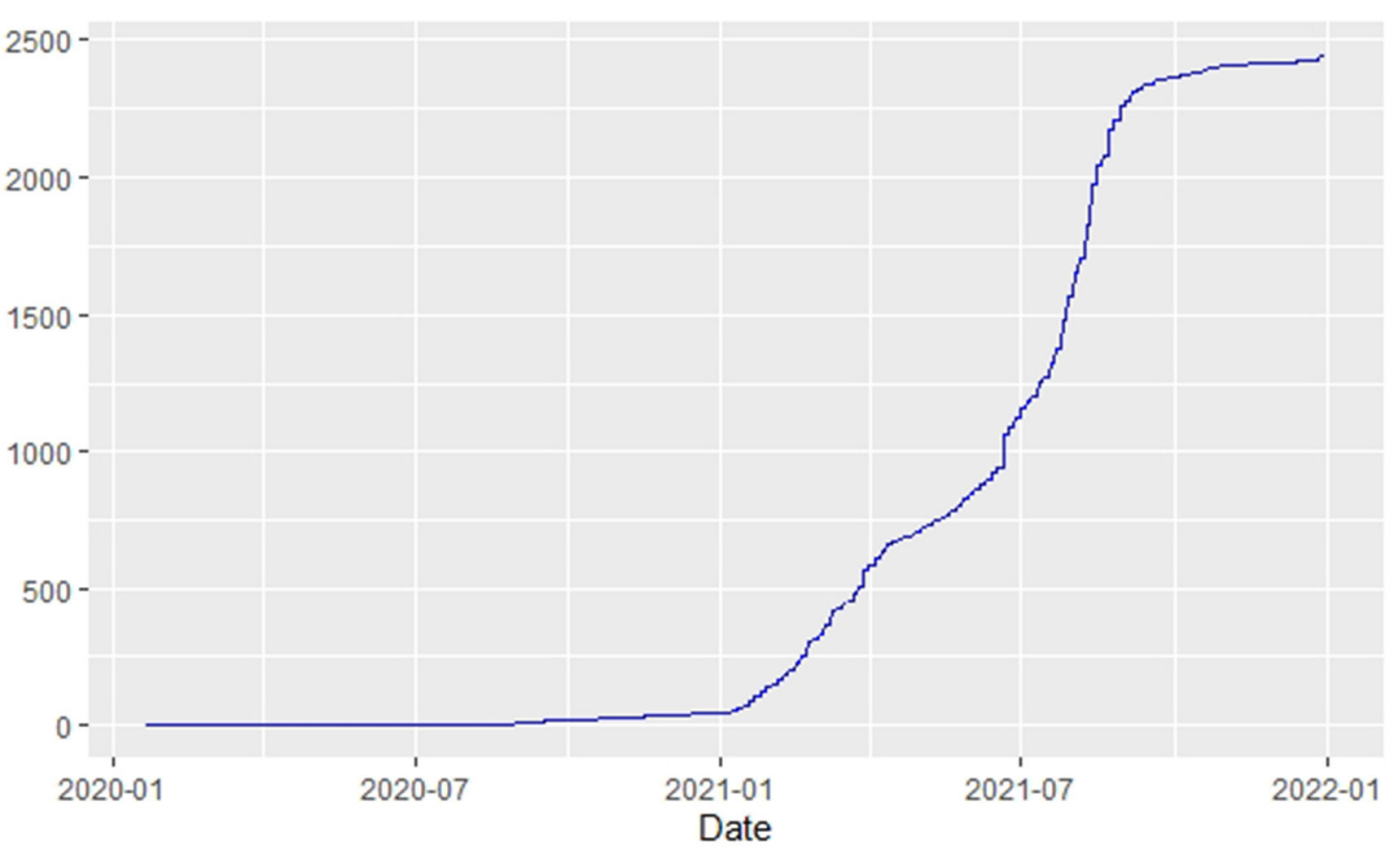
Botswana deaths.

**Figure 13:**
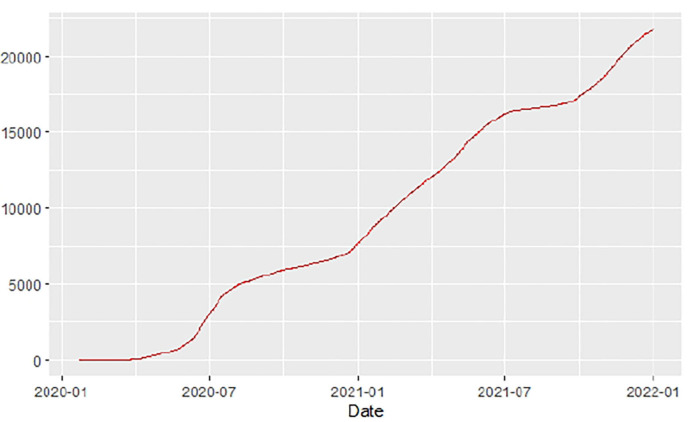
Egypt deaths.

**Figure 14:**
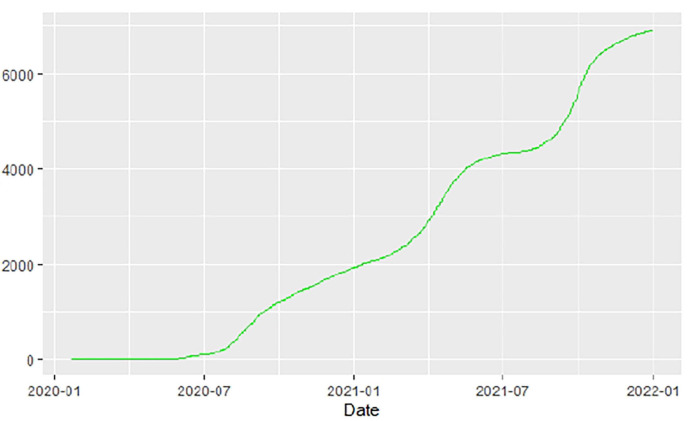
Ethiopia deaths.

**Figure 15:**
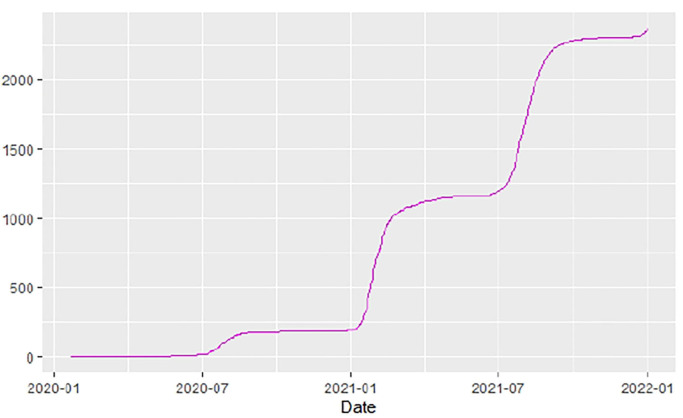
Malawi deaths.

**Figure 16:**
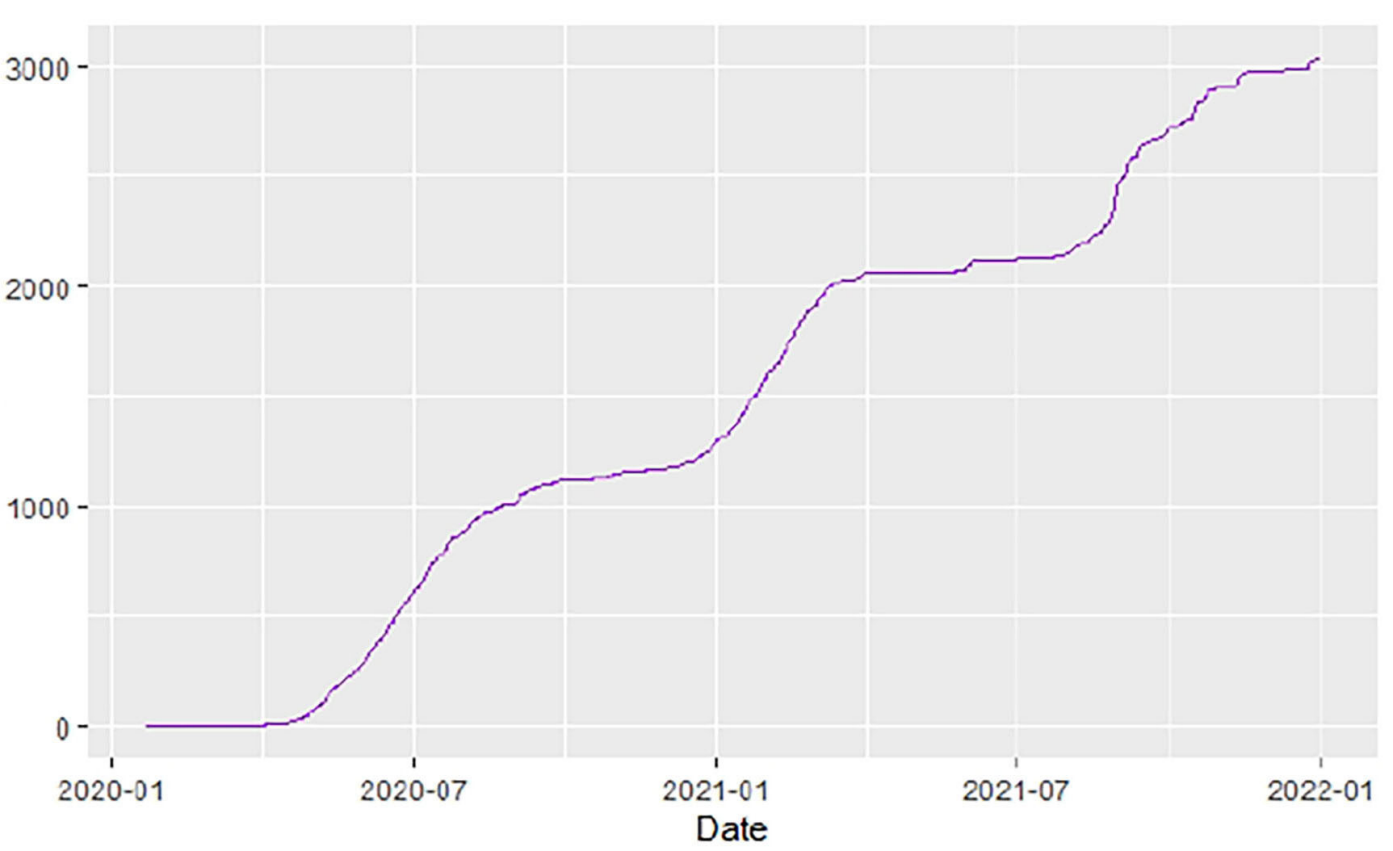
Nigeria deaths.

**Figure 17:**
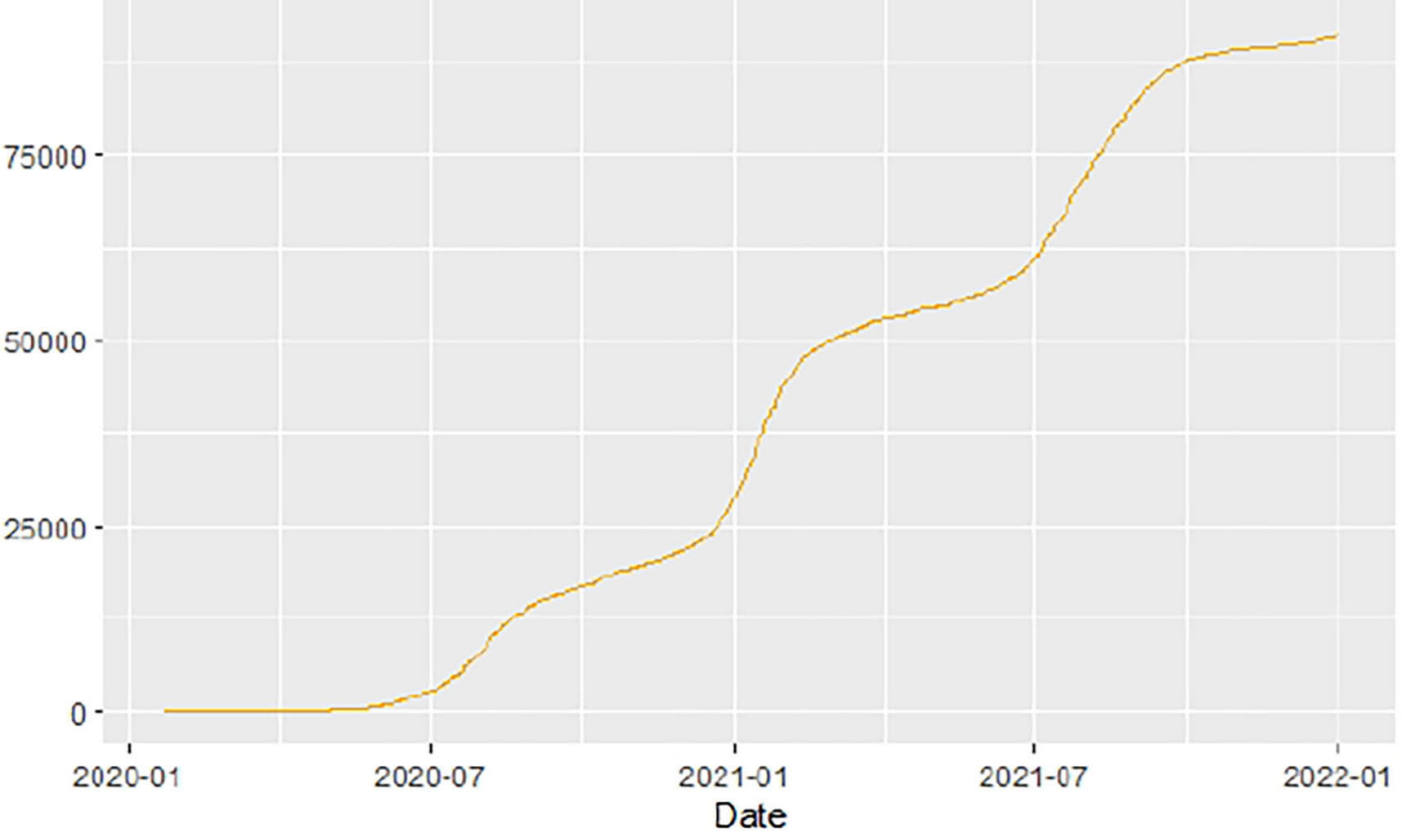
South Africa deaths.

**Figure 18:**
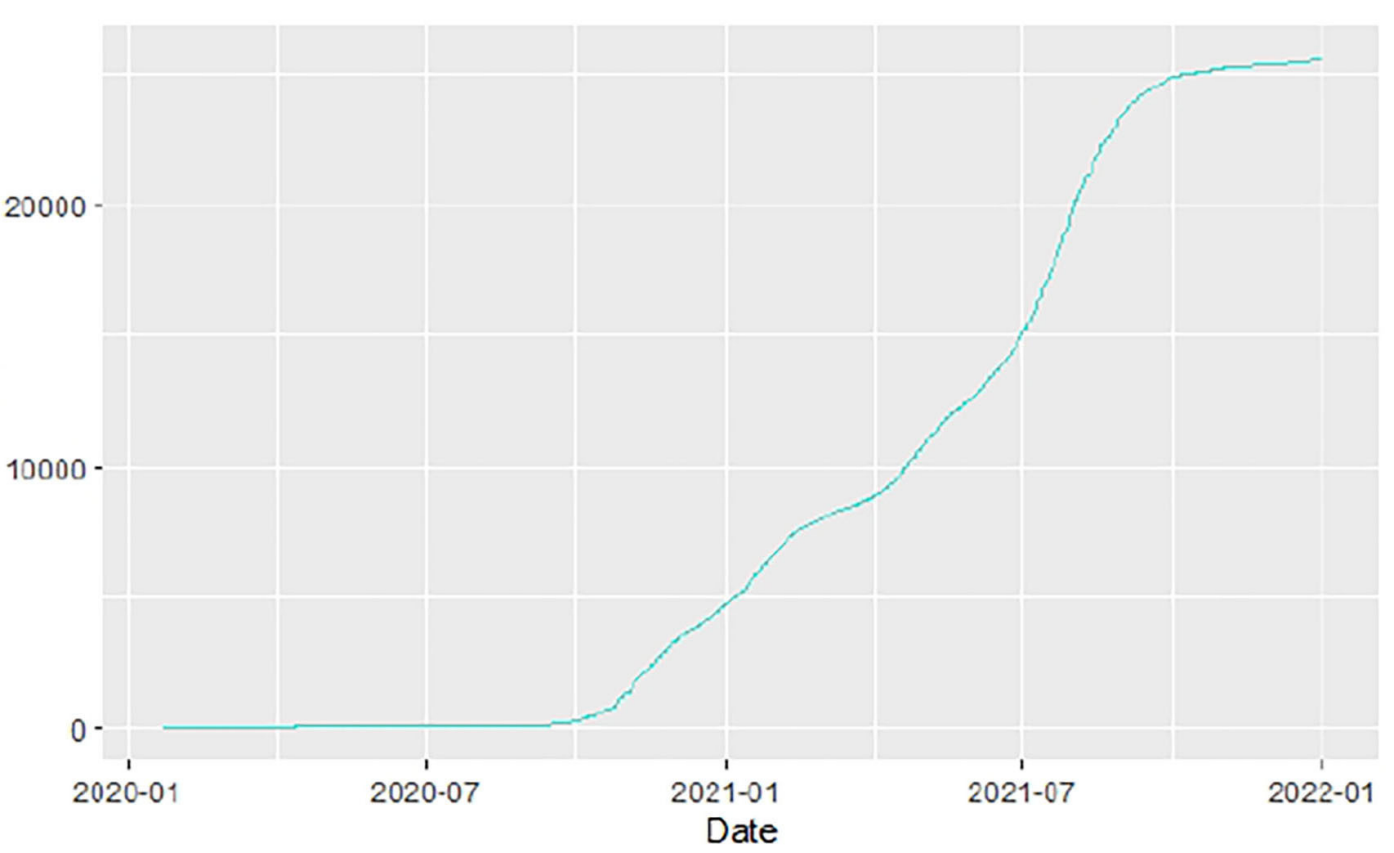
Tunisia deaths.

We then explored the epidemiological situation at the country level. We understood the linkages between COVID-19 and country-level factors for Angola, Botswana, Egypt, Ethiopia, Malawi, Nigeria, South Africa, and Tunisia to help understand the spread of the pandemic from the first day since each country registered the virus to the end of September 2021. The average case fatality rate for the selected 8 countries was observed to be 2.86%. To understand the infectivity dynamics of COVID-19 within the sampled countries, we adopted and applied a less time-consuming approach to estimating the contact rate, removal rate, basic reproduction number, infectious period, and herd immunity of COVID-19 for the 8 countries.

The results are presented in the table below. We observed the average basic reproduction number of 1.24 (vs. 1.94 in the US), contact rate of 0.61 (0.66), removal rate of 0.49(0.34), and infectious period of 2.12 (2.94) the COVID-19 pandemic in Angola, Botswana, Egypt, Ethiopia, Malawi, Nigeria, South Africa, and Tunisia, respectively, during the study period. Our analysis revealed variations in contact and removal rates, with South Africa displaying higher contact and removal rates than the other countries (Table 2). Except for Egypt, the other 6 countries showed that the contact and removal rates for COVID-19 were not different from each other. Similarly, South Africa displays a lower basic reproduction number and lower average infectious period of 1.23 days compared to the other countries. There are no significant variations in the period of infectivity and basic reproduction number for the sampled countries except for Egypt, which has a higher infectious period of 2.6 days and a basic reproduction number of 1.5869. While Egypt showed a higher herd immunity threshold, the other countries' herd immunity thresholds were not far from each other (Table 2). In 6 of the 8 countries, we found that the herd immunity threshold is less than 0.23, and in the other 2 countries, it was between 0.23 and 0.37.

**Table 2:** COVID-19 contagiousness and impact in the 8 countries.

**Country**	**Contact rate(β)**	**Removal rate(γ)**	**Basic reproduction No.(*R*_0_)**	**Infectious period ($\frac{1}{\gamma }$)**	**Herd immunity threshold (HIT)**
Angola	0.53	0.47	1.11	2.11	0.10
Botswana	0.54	0.46	1.17	2.17	0.14
Egypt	0.61	0.39	1.59	2.59	0.37
Ethiopia	0.54	0.46	1.16	2.16	0.14
Malawi	0.54	0.46	1.17	2.17	0.15
Nigeria	0.55	0.45	1.22	2.22	0.18
South Africa	1.00	0.81	1.23	1.2	0.19
Tunisia	0.56	0.44	1.30	2.30	0.23
Mean	0.61	0.49	1.24	2.12	0.20
Brazil	0.62	0.38	1.62	2.62	0.38
India	0.61	0.39	1.55	2.55	0.36
United States	0.66	0.34	1.94	2.94	0.49

**Figure 19:**
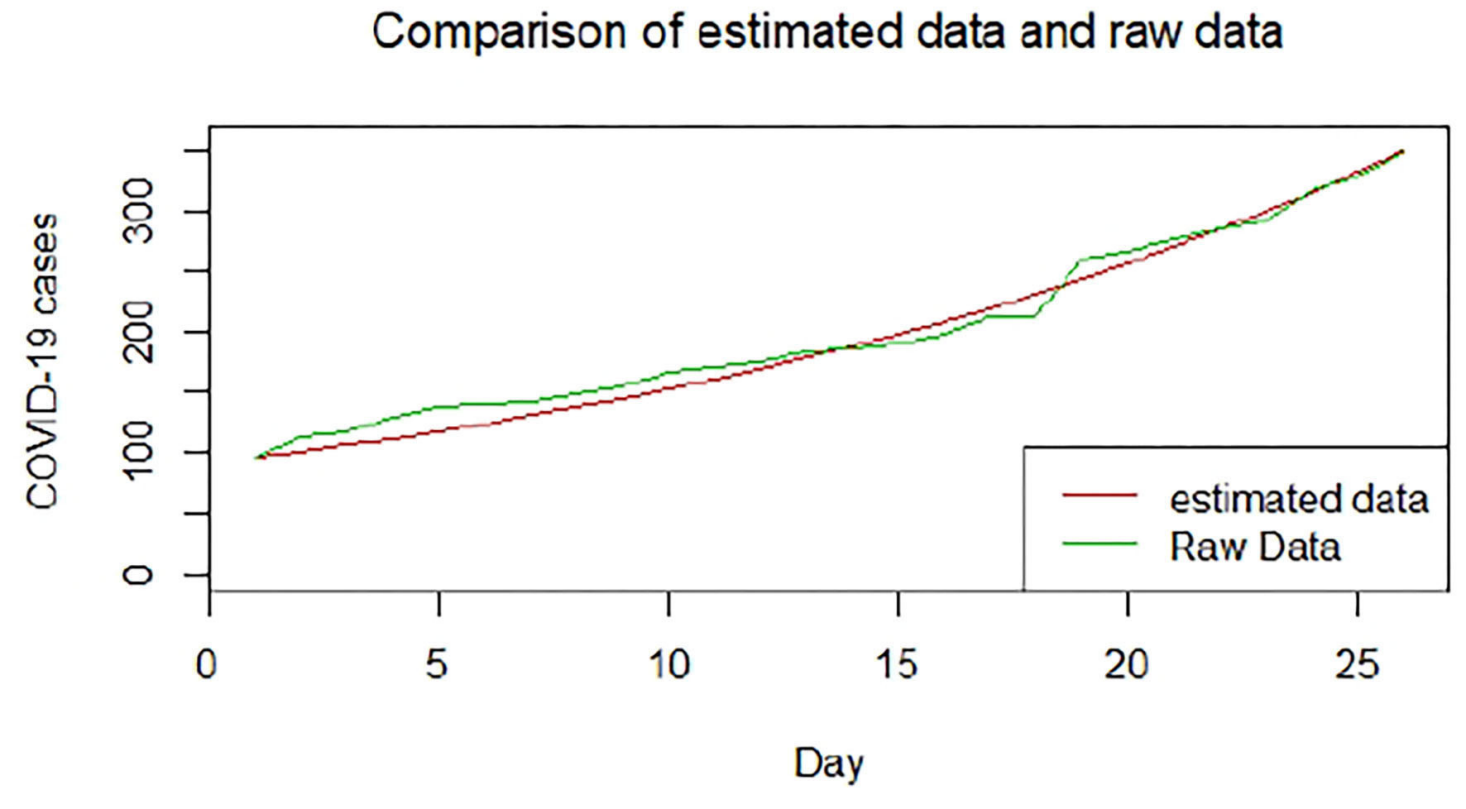
Graph for Angola.

**Figure 20:**
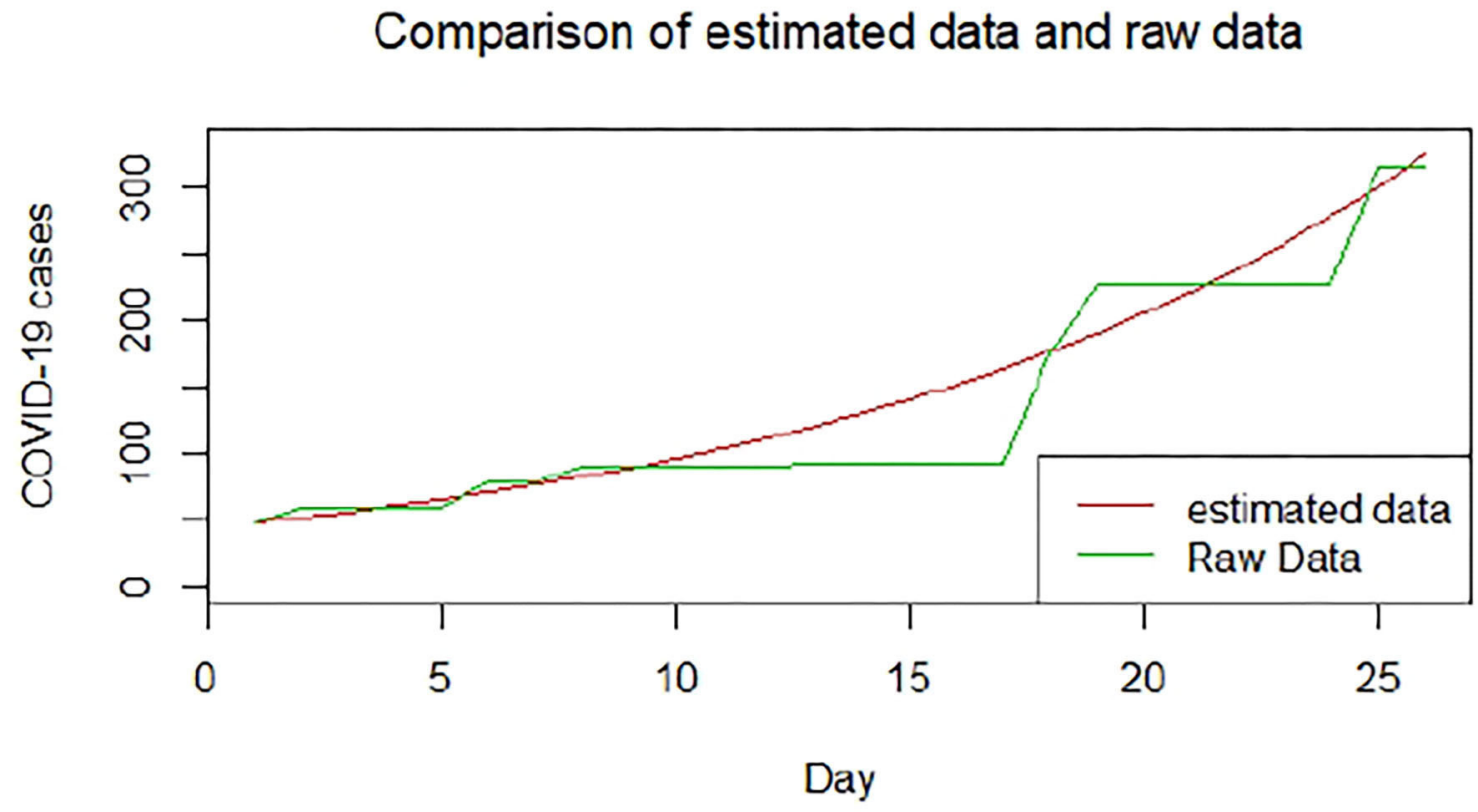
Graph for Botswana.

**Figure 21:**
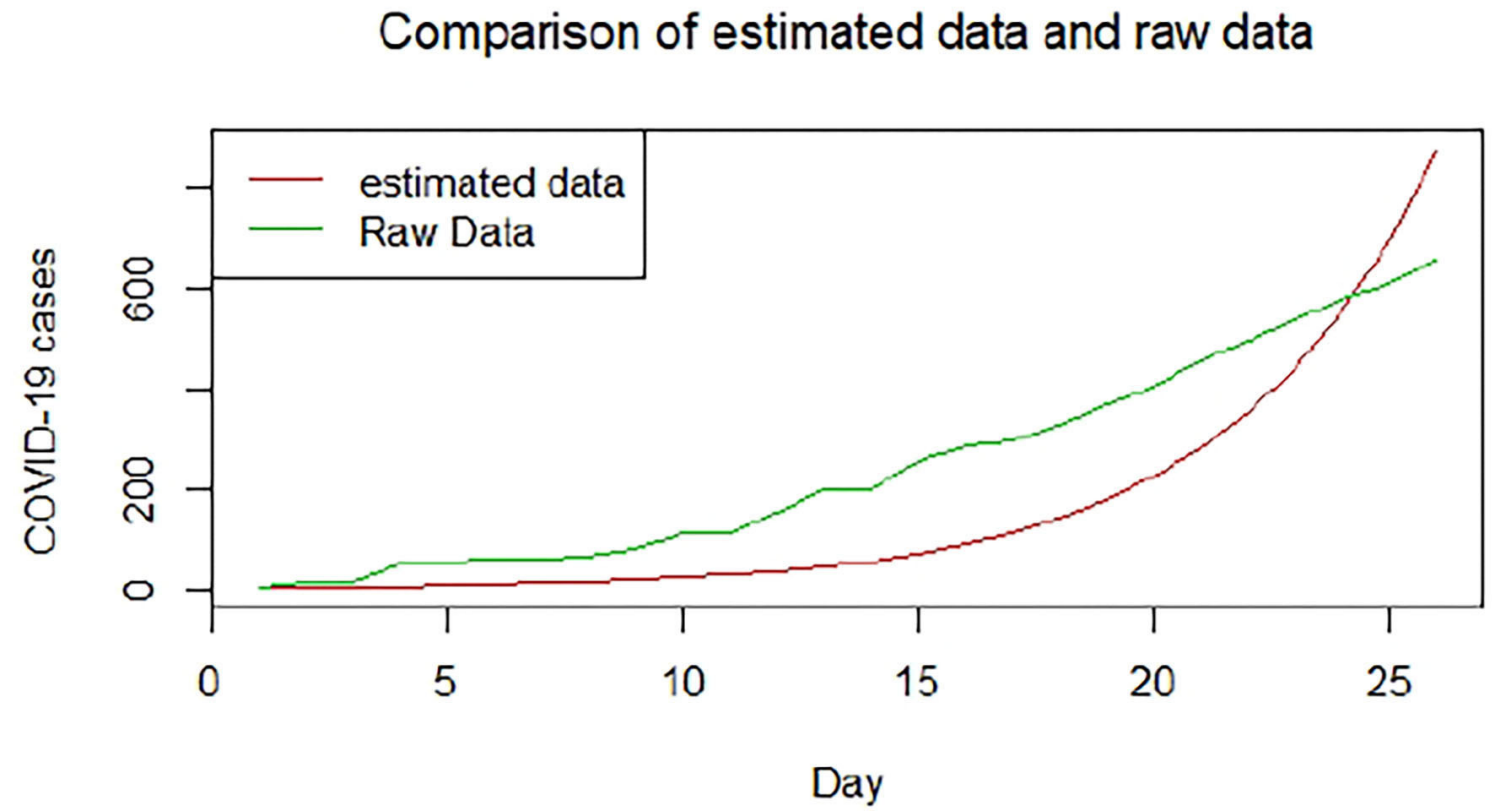
Graph for Egypt.

**Figure 22:**
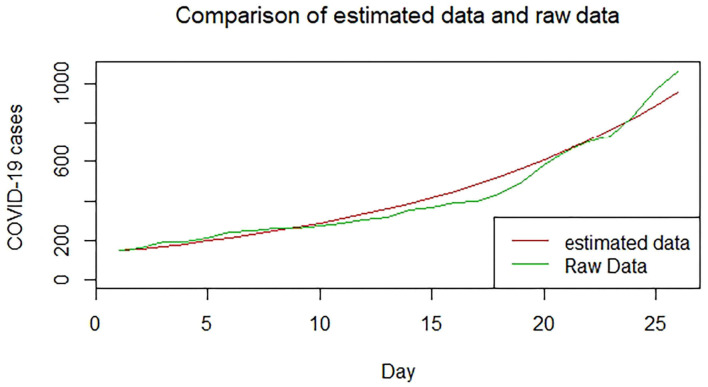
Graph for Ethiopia.

**Figure 23:**
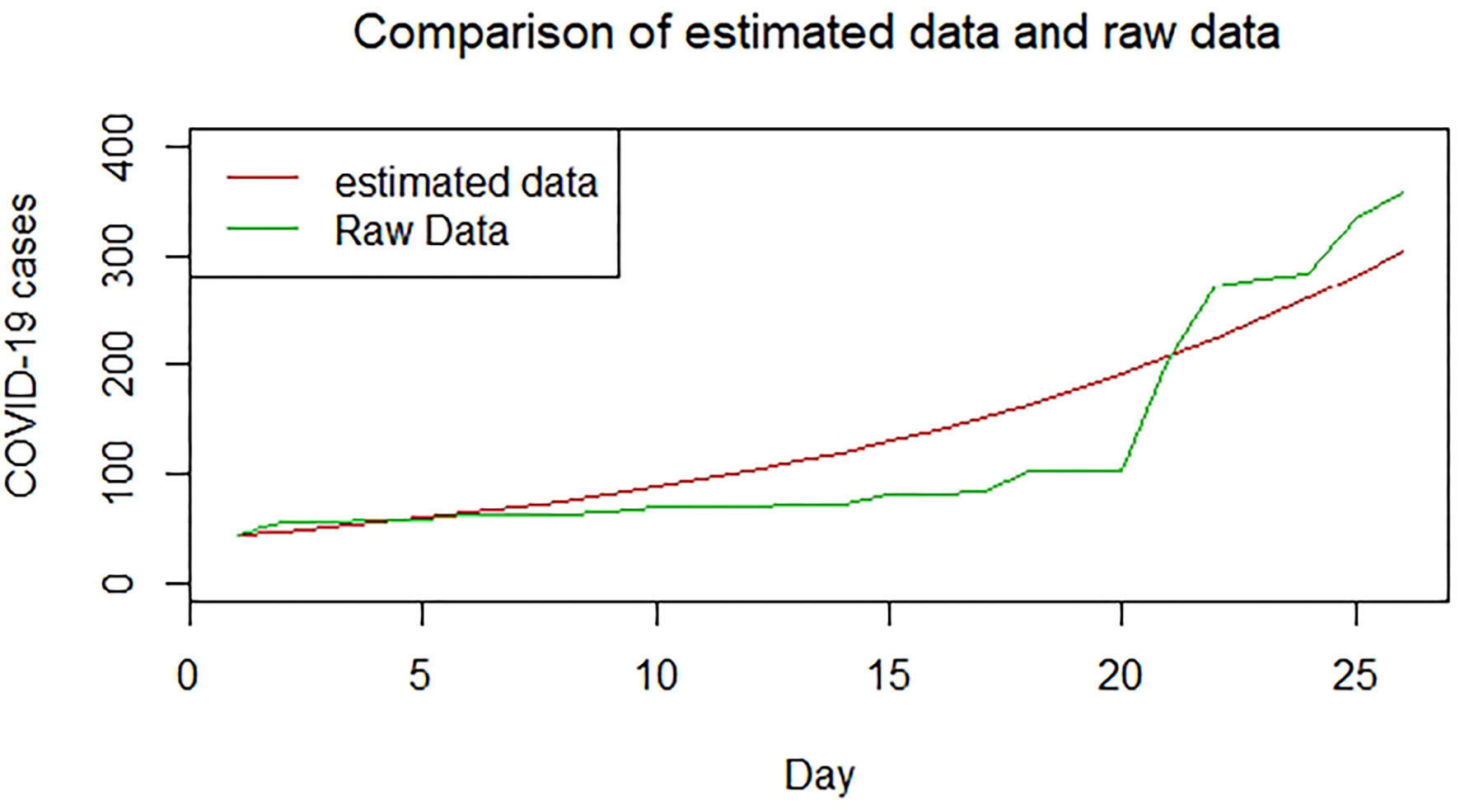
Graph for Malawi.

**Figure 24:**
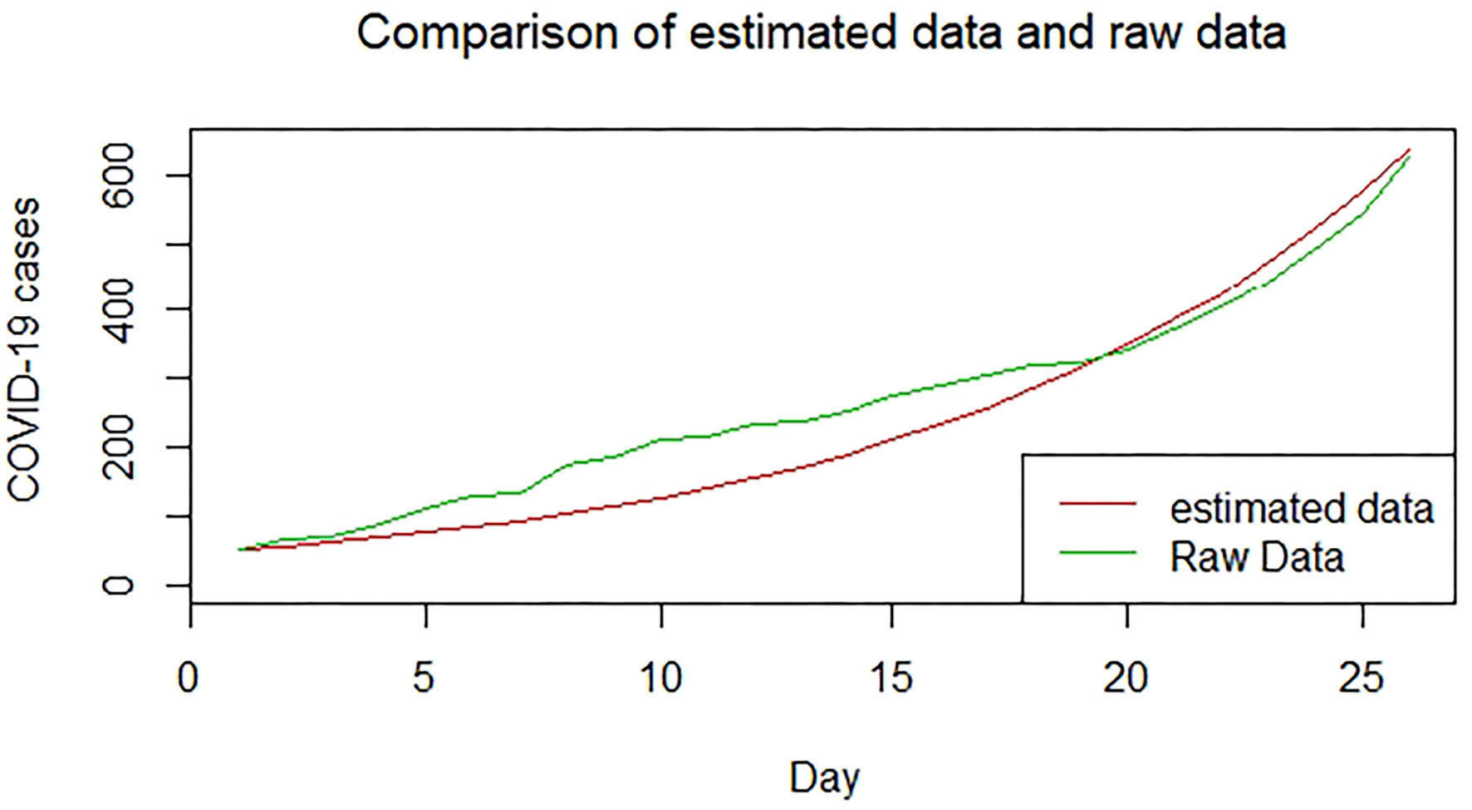
Graph for Nigeria.

**Figure 25:**
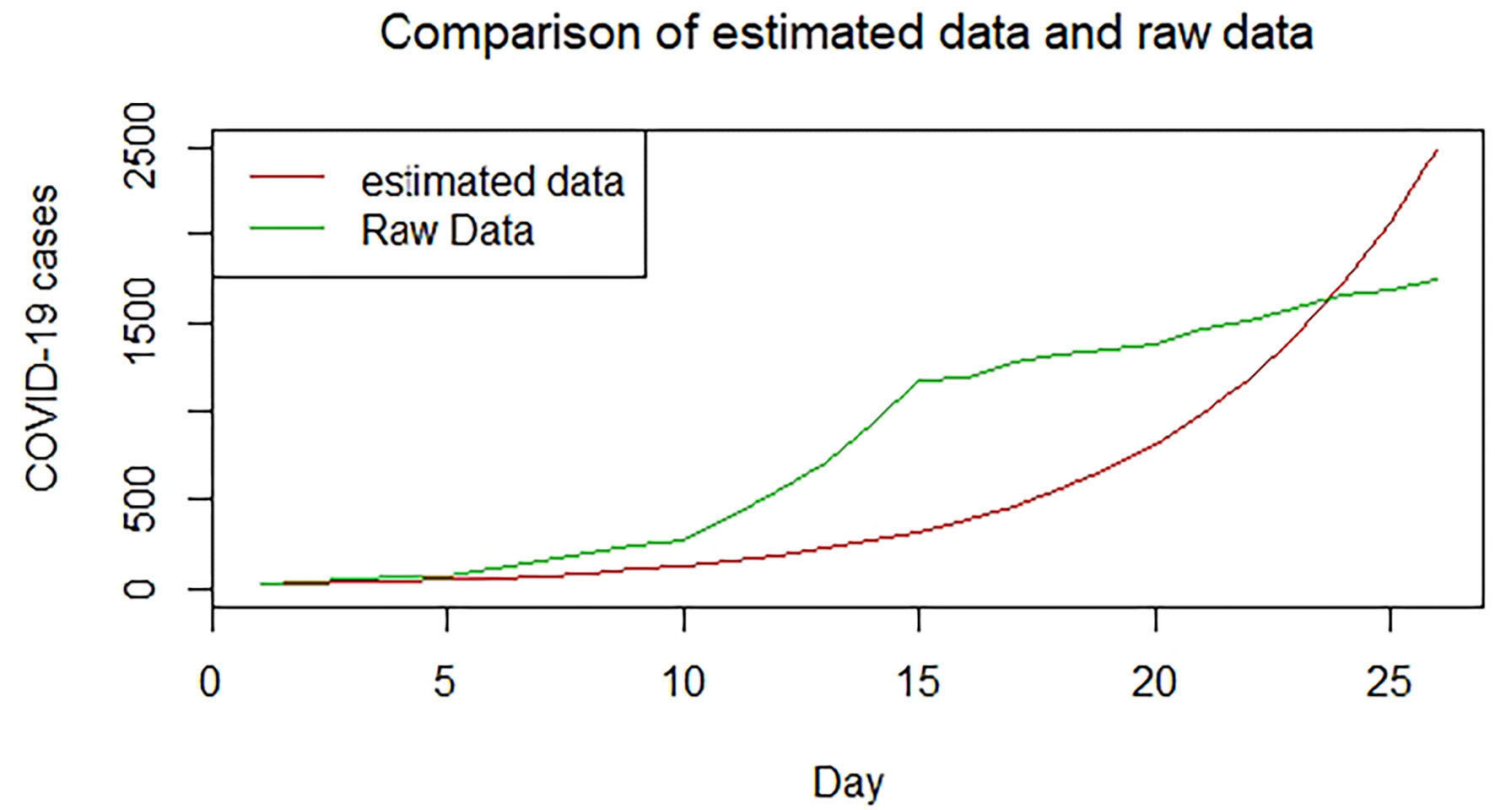
Graph for South Africa.

**Figure 26:**
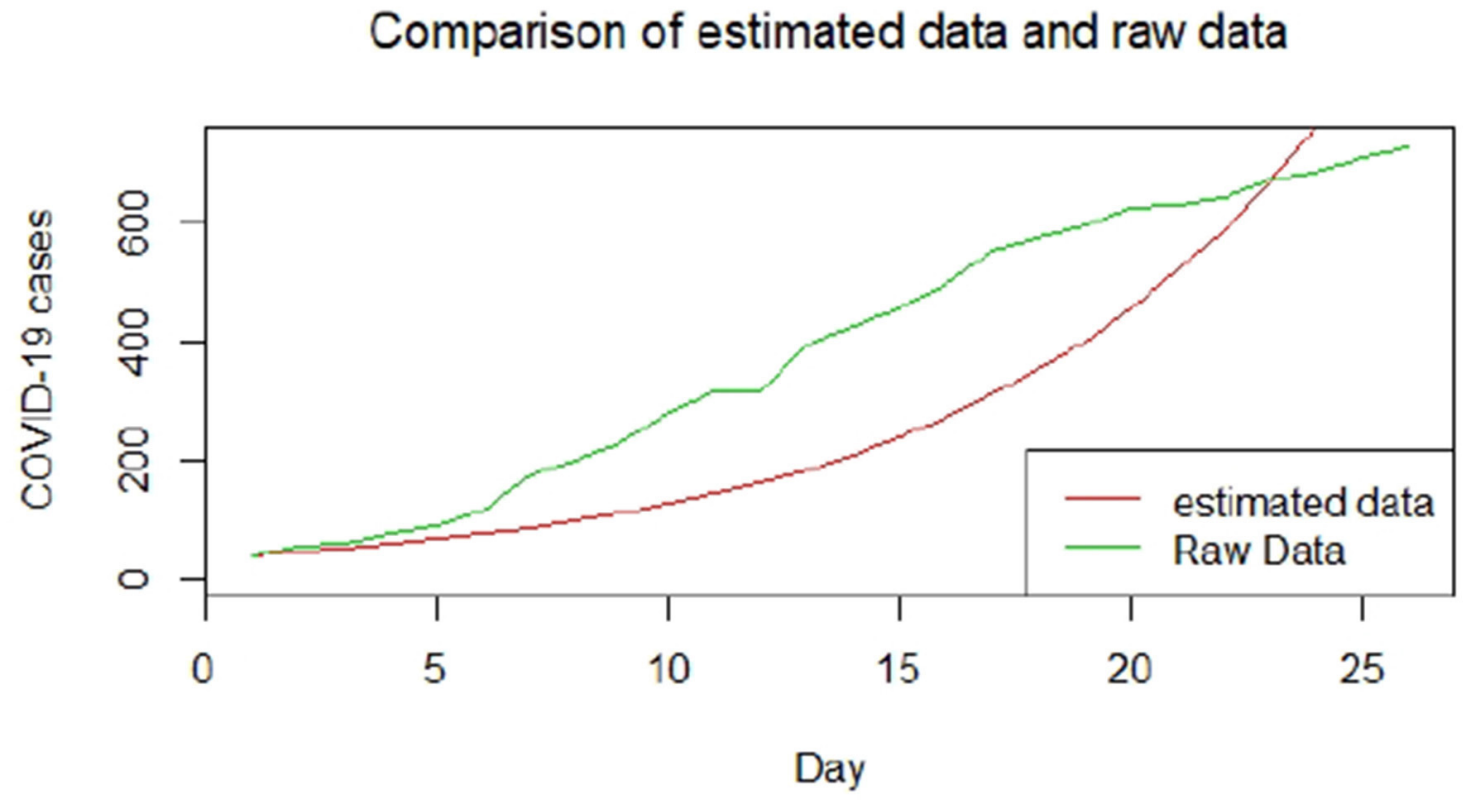
Graph for Tunisia.

We plotted the first 25 observed confirmed cases against the respective predicted cases to understand model fit. Figures 19–26 show the comparison between predicted confirmed cases and observed for the 8 sampled African countries for the first 25 days since each country registered an active case. Based on the Angola graph, the model almost fitted the predicted and actual values well, with minor underestimation observed for the first 15 days. Ethiopia also better fitted the predicted values for the first 10 days and the period between days 20 and 25. The model then overestimated the predicted values between days 10 and 20. While Botswana shows a good fit between the predicted and actual values for the first 10 days, a similar case was observed for the first 8 days for Malawi. The Botswana and Malawi graphs overestimated the predicted values between the 10th day to the 18th day and the 10th day to the 20th day, respectively. Plots for Egypt, Nigeria, South Africa, and Tunisia consistently show that the model underestimated the predicted values until later when it overestimated COVID-19 cases.

To assess associations between the estimated basic reproduction number, contact rate, and removal rate and select 20 COVID-19 vulnerability factors, we performed pairwise correlation analyses. Table 3 shows the COVID-19 vulnerability factors and their associations with the three epidemiological parameters. We had set 0.05 as a statistically significant level due to the problems of multiple comparisons as a result of testing 20 correlations for significance with each of the three epidemiological parameters. Thus, we conservatively adjusted the significant level to 0.05/20=0.0025. Only contact rate and removal rate had a significant correlation with total COVID-19 deaths (*p* < 0.0025).

**Table 3:** Association of reproduction number, contact rate, and removal rate with socioeconomic determinants.

	**Reproduction number correlation (*p*-value)**	**Contact rate correlation (*p*-value)**	**Removal rate correlation (*p*-value)**
Total population	–0.19 (0.65)	–0.02 (0.97)	–0.10(0.82)
Percentage of female	–0.34 (0.41)	0.11 (0.79)	0.30 (0.47)
GDP per capita	–0.19 (0.65)	0.39 (0.34)	0.26 (0.53)
Proportion aged ≥ 65 yrs	–0.02 (0.97)	0.33 (0.42)	0.12 (0.78)
Daily COVID-19 case fatality rate	–0.14 (0.75)	0.17 (0.69)	–0.12(0.79)
Density (P/sq. KM)	0.24 (0.57)	–0.24(0.57)	–0.27 (0.51)
Fertility Rate	0.24 (0.57)	–0.49 (0.21)	–0.34 (0.41)
Median age	–0.17 (0.69)	0.45 (0.27)	0.26 (0.53)
Total deaths due to COVID-19	–0.82 (0.01)	0.98 (0.001)^*^	0.89 (0.003)
Proportion of people who received ≥ 1 dose	0.41 (0.31)	0.27 (0.52)	0.18 (0.67)
Prevalence of diabetes	0.41 (0.31)	0.10 (0.82)	–0.24 (0.56)
Mortality rate due to cardiovascular	0.55 (0.16)	–0.09 (0.82)	–0.41 (0.31)
International exposure	–0.18 (0.67)	0.50 (0.21)	0.27 (0.51)
Public health systems	–0.08 (0.85)	–0.28 (0.50)	–0.05 (0.90)
Density of urban areas	0.37 (0.36)	–0.39 (0.35)	–0.39 (0.34)
Total populations in urban areas	–0.02 (0.97)	0.22 (0.60)	0.08 (0.86)
Government transparency	0.36 (0.38)	–0.43 (0.29)	–0.41 (0.31)
Press freedom	0.73 (0.04)	–0.68 (0.06)	–0.73 (0.04)
Conflict magnitude	0.26 (0.54)	–0.24 (0.57)	–0.28 (0.51)
Forced displacement	–0.12 (0.78)	0.20 (0.63)	0.14 (0.74)

* *significant at p-value = 0.0025*.

## 4. Discussion

Using publicly available COVID-19 data for the period 1 January 2020 to 31 December 2021, this study has estimated basic reproduction number, contact rate, and removal rates for COVID-19 in eight African countries using the classical *SIR* model. The study also compared and validated our approach using COVID-19 for the same period in the USA, Brazil, and India which are some of the countries with the most COVID-19 outbreaks. The study found positive significant associations between contact and removal rates with total deaths due to COVID-19. Thus, for the studied countries, higher removal rates could not have been attributed to patients recovering (and hence gaining immunity) but more to those who have died from the disease. Our findings have also revealed a higher herd immunity threshold for Egypt and Tunisia, respectively. Thus, these two countries would require higher rates of 60% or more COVID-19 vaccinations than in the other six African countries (30). Our study found variations in contact rates between South Africa and the other seven countries. Our findings compare well with those (31) who found differences in the contagiousness of COVID-19 between countries. The differences could be attributed to differences in COVID-19 vulnerability factors such as social behavior and political strategies (19, 20). South Africa was found to have a high removal rate than the other countries, suggesting a better rate of recovery of patients from COVID-19. Also, South Africa had the fastest spread of COVID-19 at 1.2 days.

Our findings could be subjected to some limitations that could have influenced our results based on the assumptions of our modeling approach. Our SIR model approach assumes a homogeneous population, a constant rate of infections, and a non-quantification of uncertainty from model parameters. Moreover, the model does not incorporate the latent period between when an individual is exposed to a disease and becomes infected and contagious (17, 32). In this study's context, these assumptions may be limiting factors as the vulnerability of countries differs and vary with continuous changes in population due to migration, births, and deaths, which directly affect COVID-19 testing and vaccinations.

## 5. Conclusion

We applied a basic SIR mathematical model to understand of COVID-19 epidemic in eight African countries. The insights drawn from this study could be vital in understanding how regional coordinated efforts could play a critical role in containing the pandemic. However, the limitation lies in knowing the precise basic reproduction number which is directly linked to the precision of the data and its quality. Although, the simple SIR model could not have been sufficient to test the effects of different interventions that could be fit to understand the dynamics of the COVID-19 epidemiology, including Omicron and other potentially novel emerging SARS-CoV-2 variants.

## Data Availability Statement

Publicly available datasets were analyzed in this study. This data can be found here: https://raw.githubusercontent.com/CSSEGISandData/COVID-19/master/csse_covid_19_data/csse_covid_19_time_series/time_series_covid19_confirmed_global.csv.

## Ethics Statement

Our study utilized publicly and anonymised aggregated COVID-19 case and death data. Thus, individual consent to participate did not apply. Ethical review and approval was not required for the study on human participants in accordance with the local legislation and institutional requirements. Written informed consent for participation was not required for this study in accordance with the national legislation and the institutional requirements.

## Author Contributions

GS performed data management, statistical analysis, and wrote the initial draft of the manuscript. SOMM conceived ideas for this article, reviewed statistical analysis and results, and helped with the revision of the manuscript. Both authors have read and approved the final revised manuscript.

## Funding

SM was supported by the South African Medical Research Council.

## Conflict of Interest

The authors declare that the research was conducted in the absence of any commercial or financial relationships that could be construed as a potential conflict of interest.

## Publisher's Note

All claims expressed in this article are solely those of the authors and do not necessarily represent those of their affiliated organizations, or those of the publisher, the editors and the reviewers. Any product that may be evaluated in this article, or claim that may be made by its manufacturer, is not guaranteed or endorsed by the publisher.
