# Supplementary figures and images for: Inter-Country COVID-19 Contagiousness Variation in Eight African Countries
Source: Front Public Health. 2022 Jun 2;10:796501. doi: 10.3389/fpubh.2022.796501 (PMC9201645; doi:10.3389/fpubh.2022.796501)

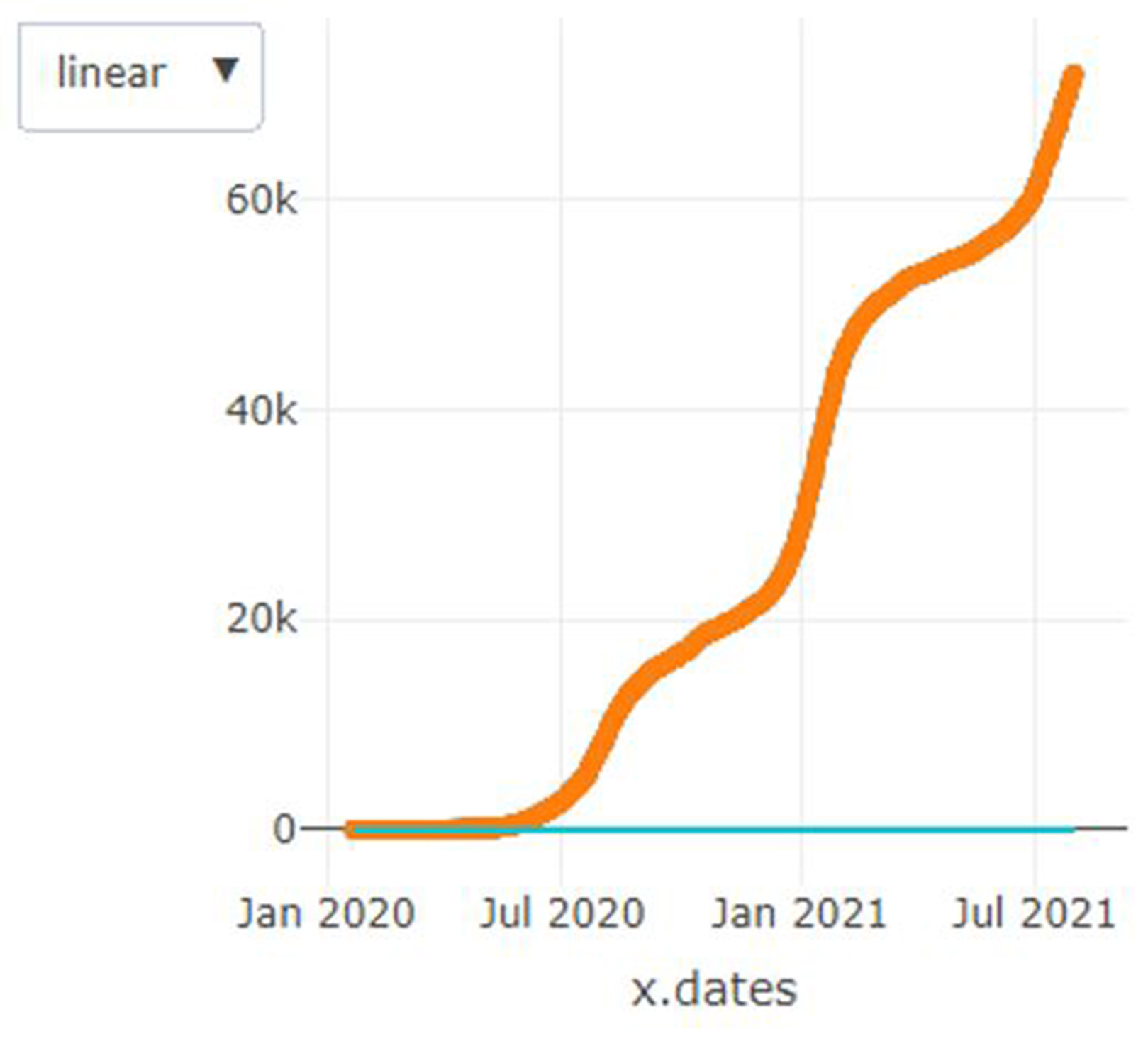

Supplement: Supplementary file 1 [file Image_1.JPEG]

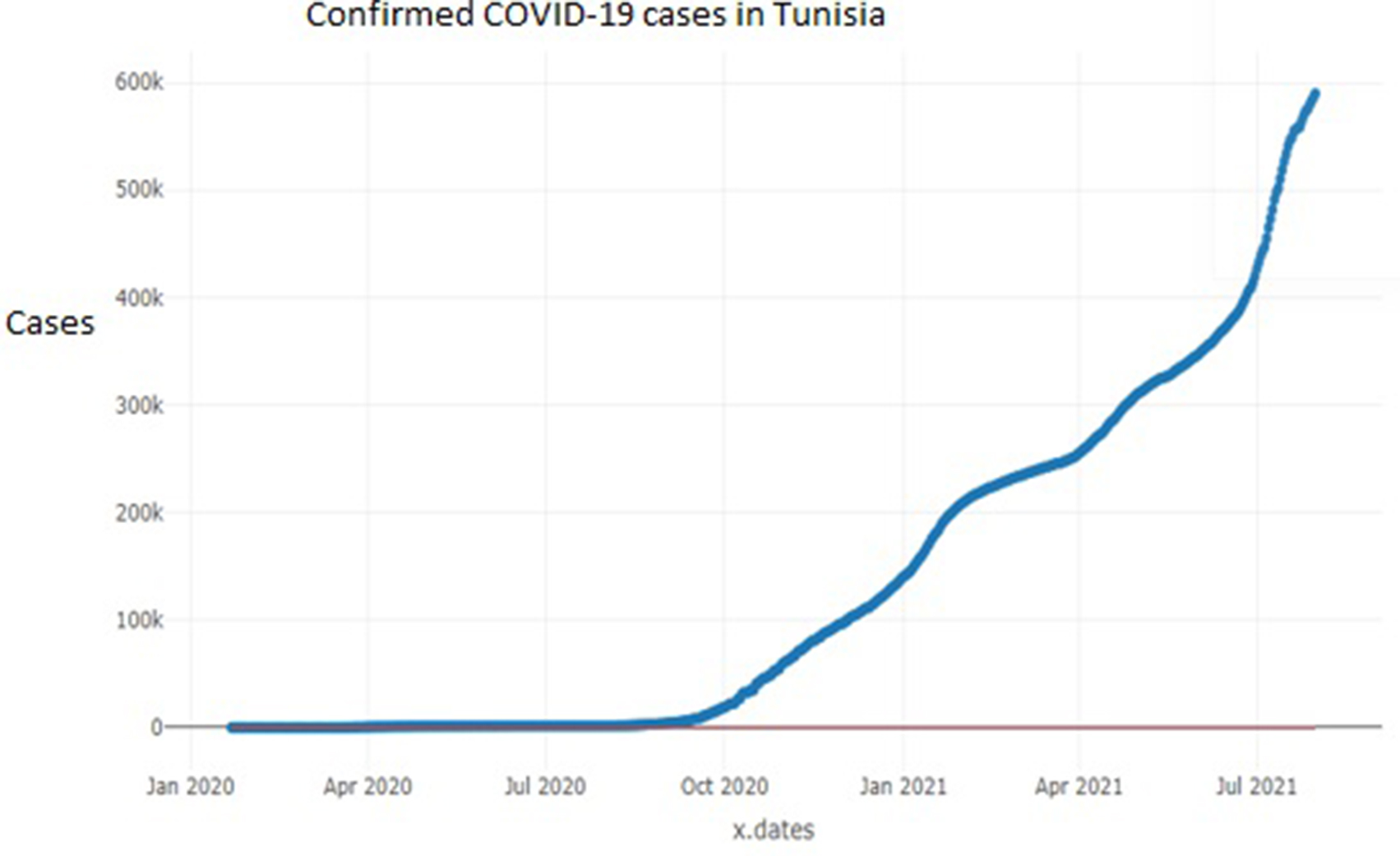

Supplement: Supplementary file 2 [file Image_2.JPEG]

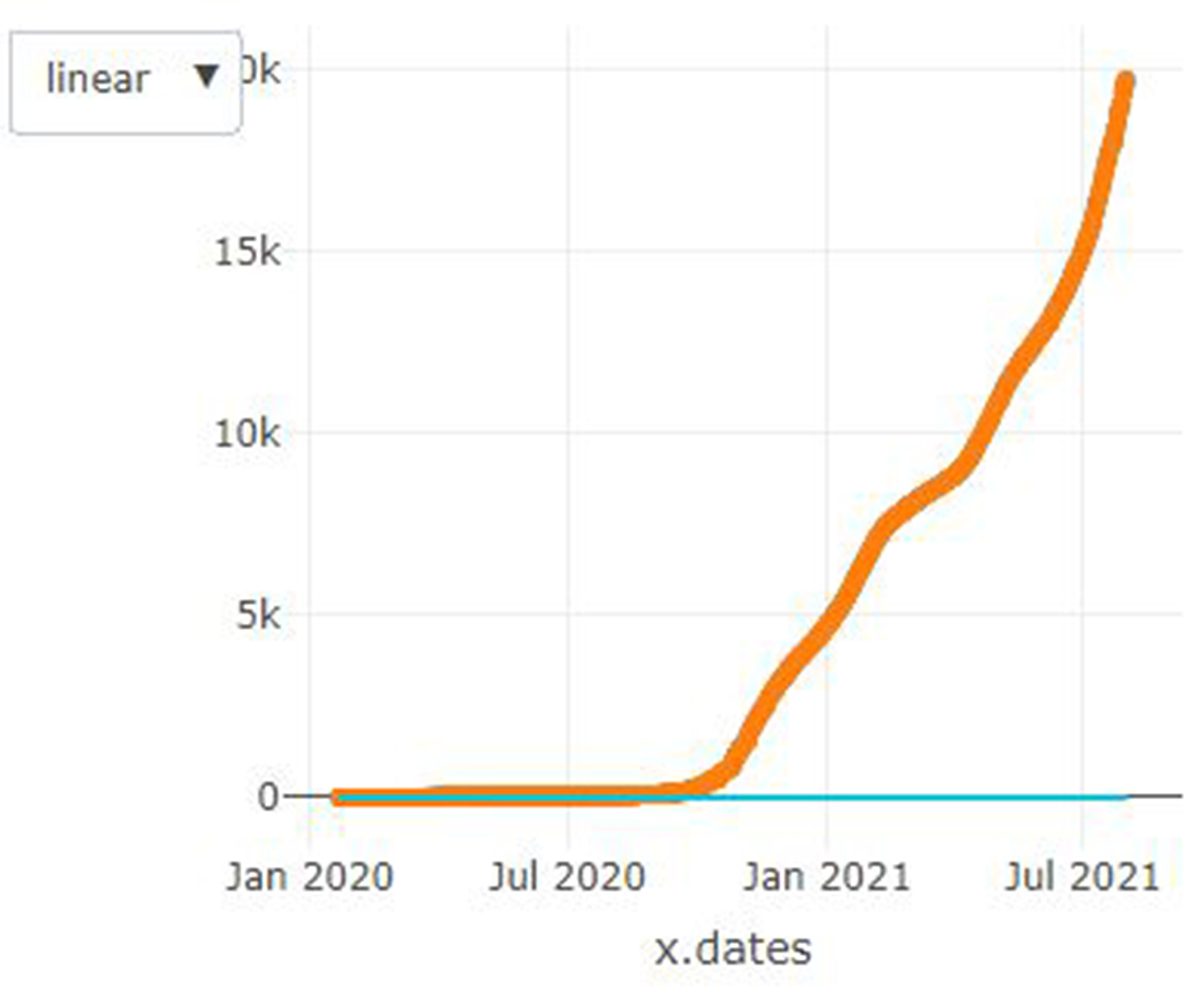

Supplement: Supplementary file 3 [file Image_3.JPEG]

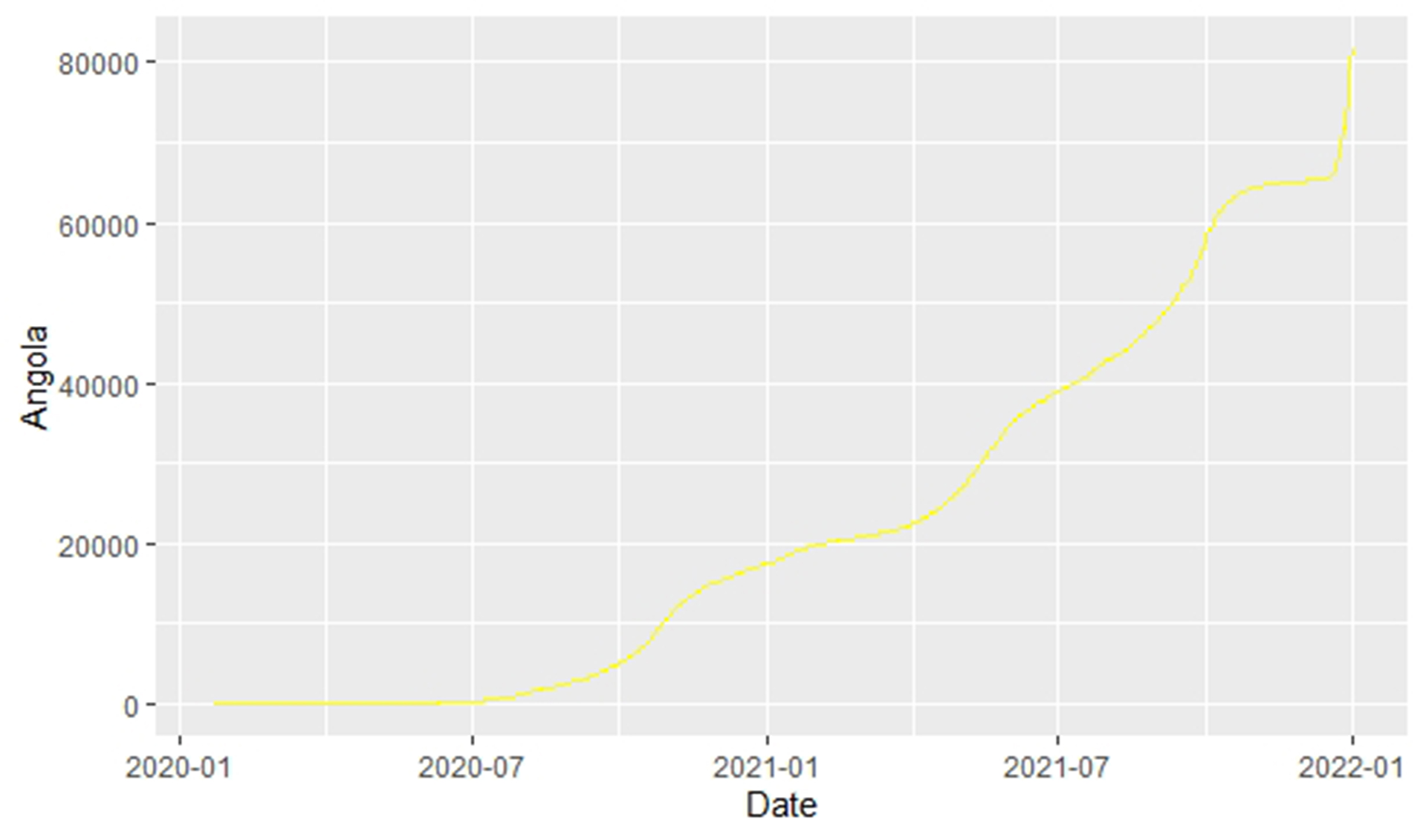

Supplement: Supplementary file 4 [file Image_4.JPEG]

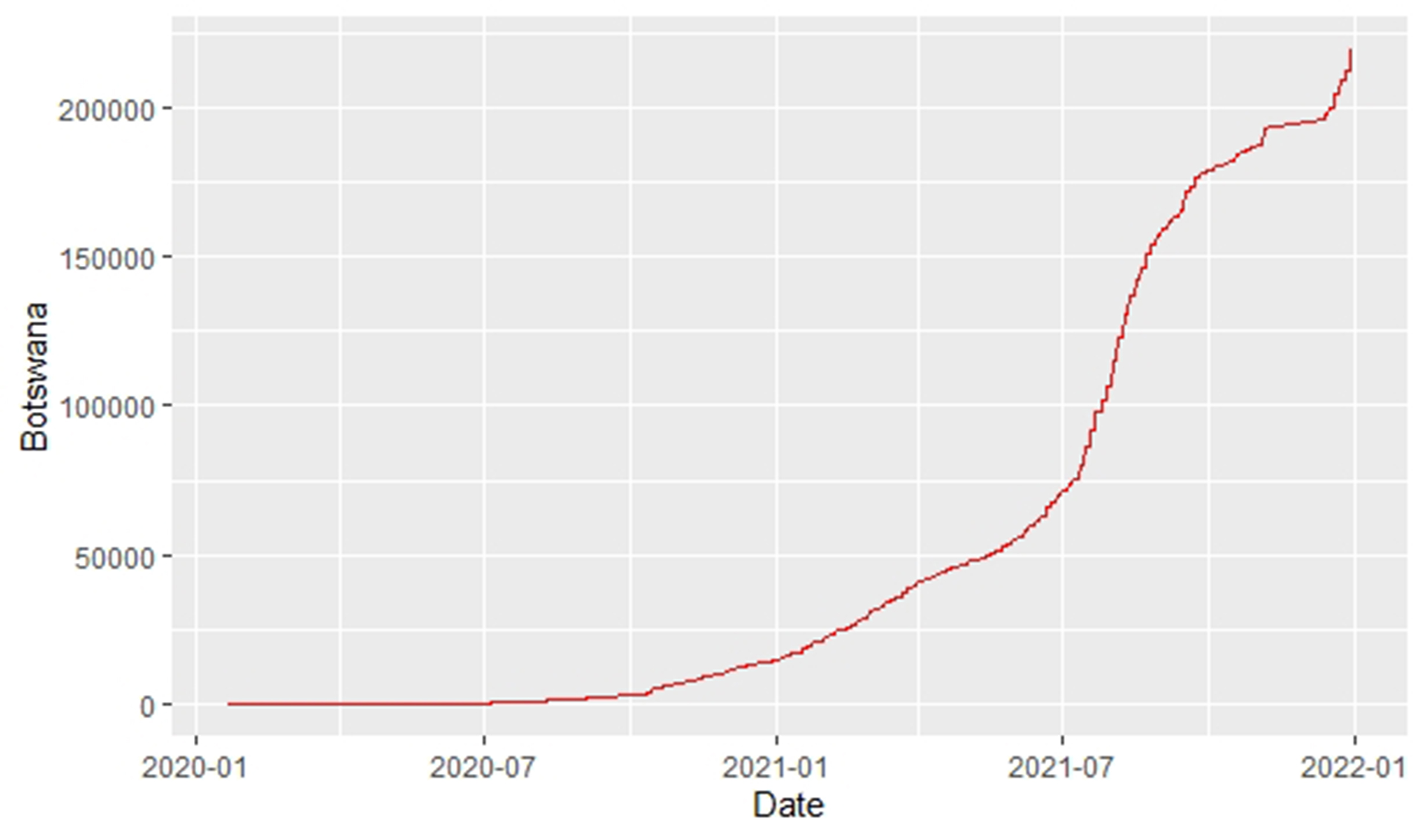

Supplement: Supplementary file 5 [file Image_5.JPEG]

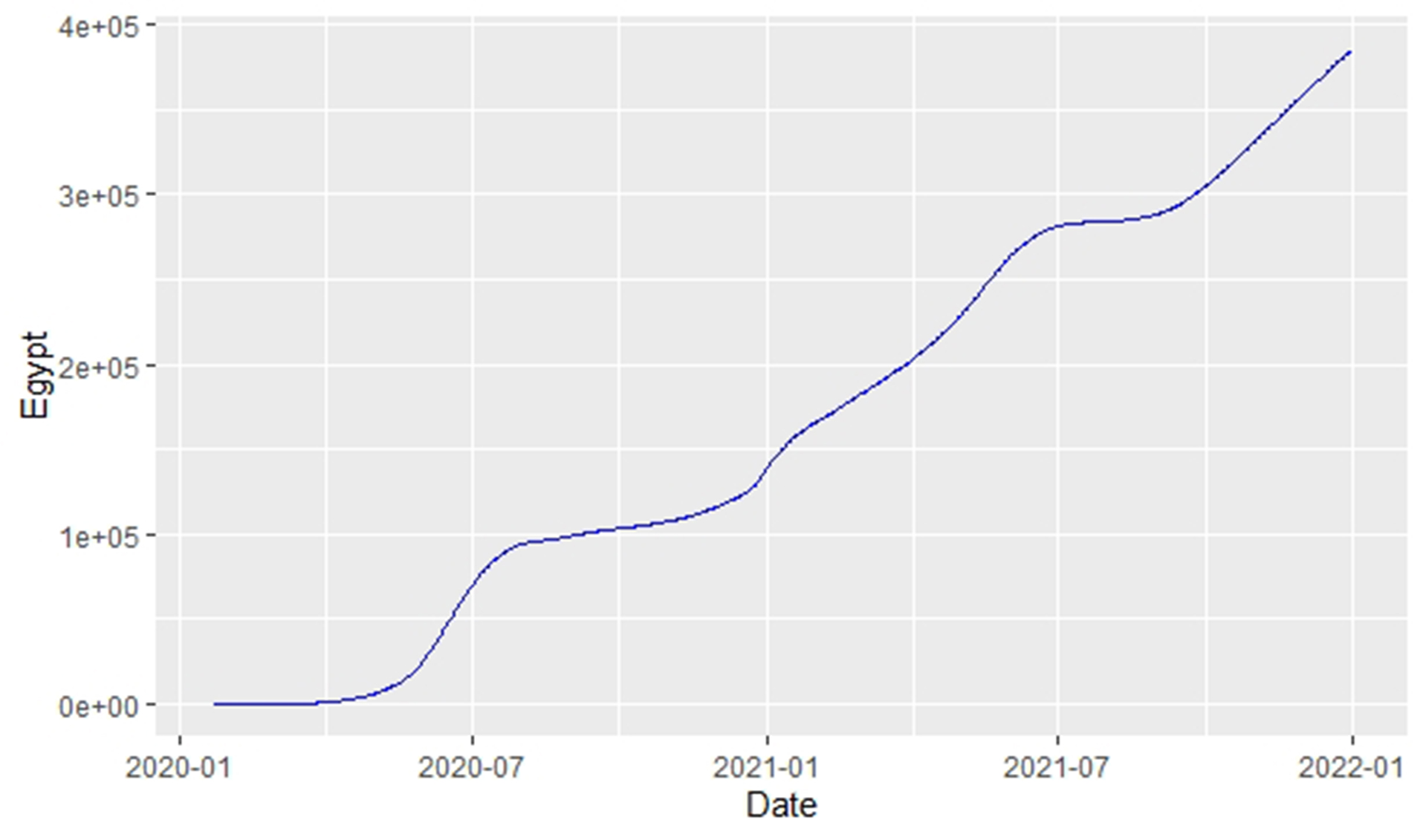

Supplement: Supplementary file 6 [file Image_6.JPEG]

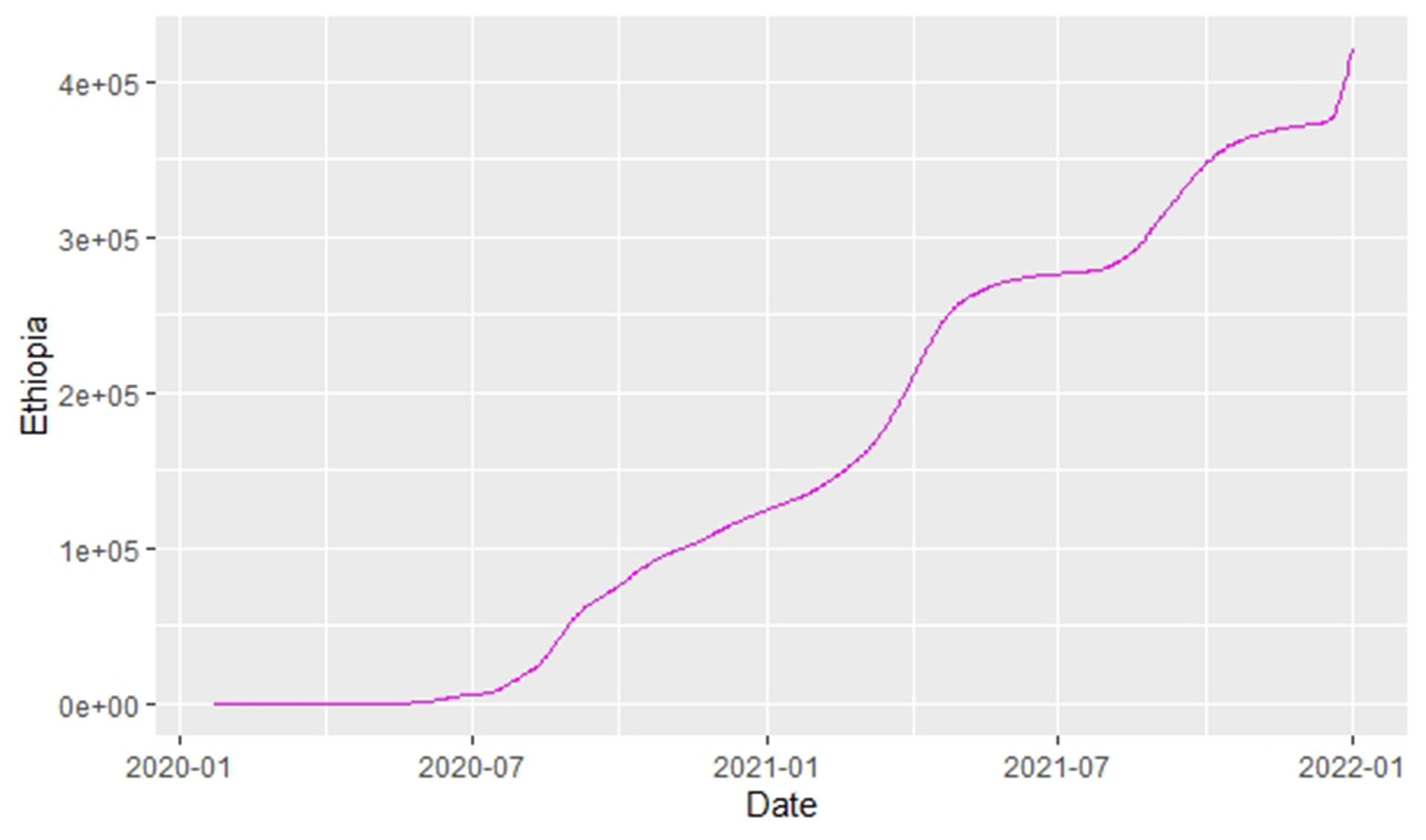

Supplement: Supplementary file 7 [file Image_7.JPEG]

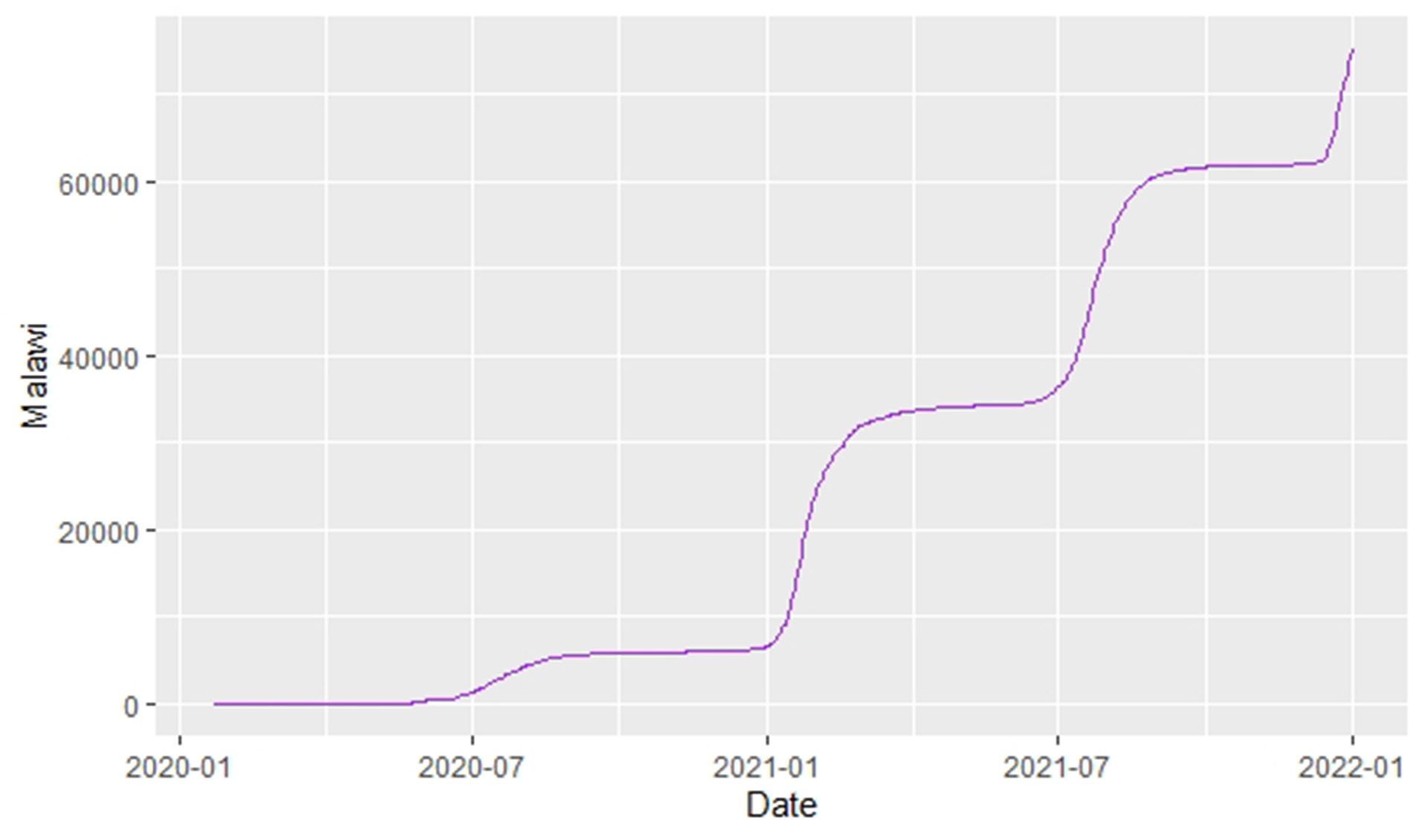

Supplement: Supplementary file 8 [file Image_8.JPEG]

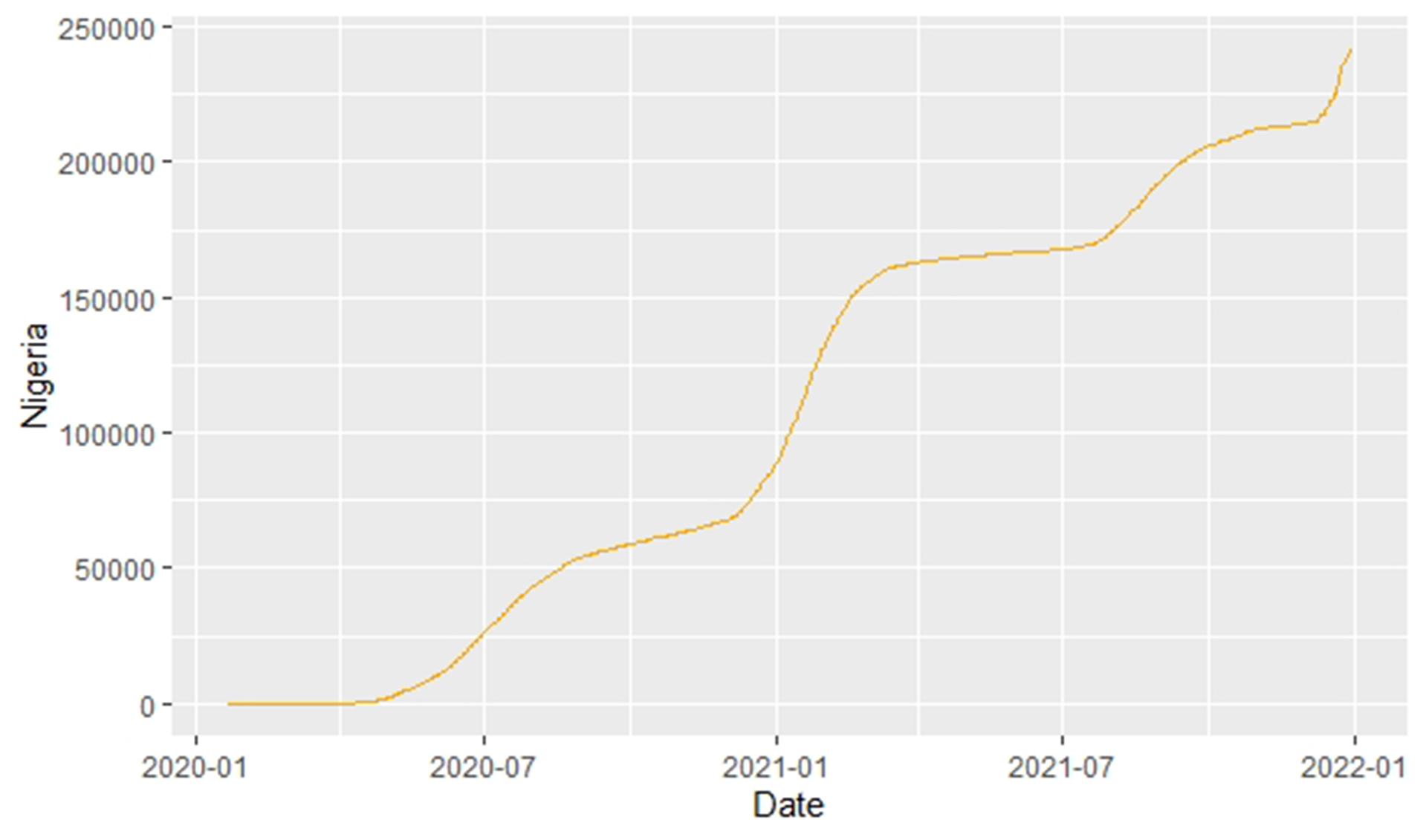

Supplement: Supplementary file 9 [file Image_9.JPEG]

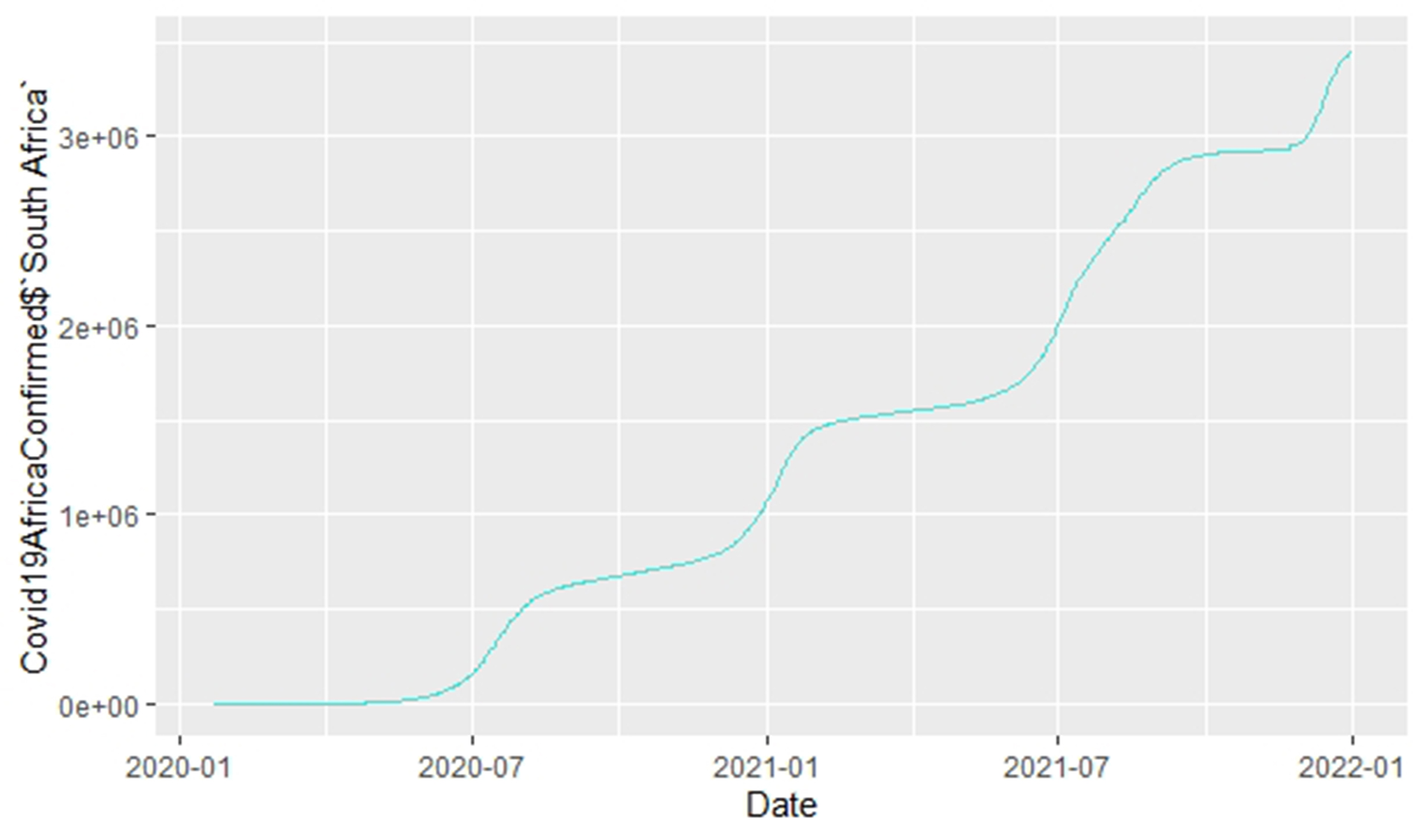

Supplement: Supplementary file 10 [file Image_10.JPEG]

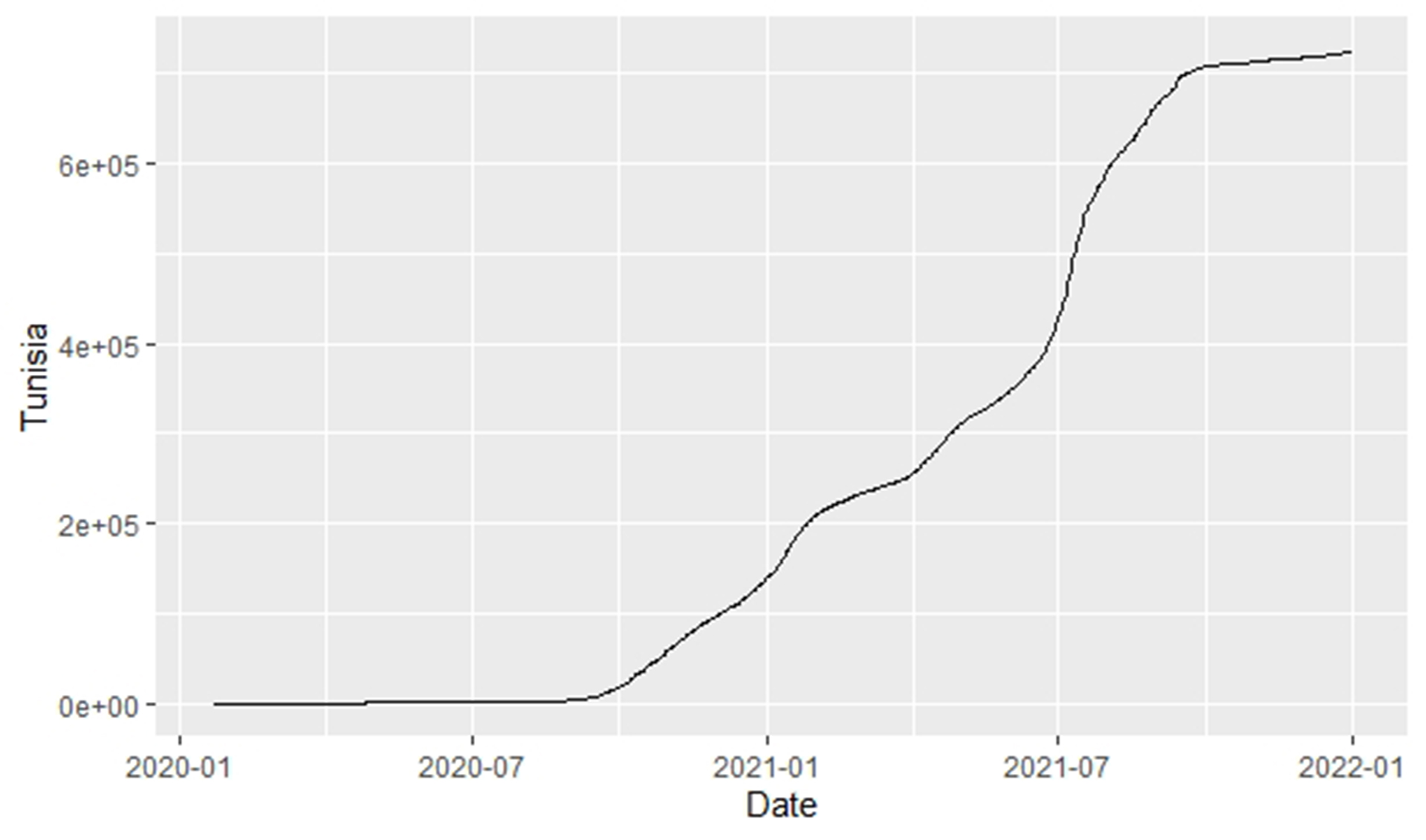

Supplement: Supplementary file 11 [file Image_11.JPEG]

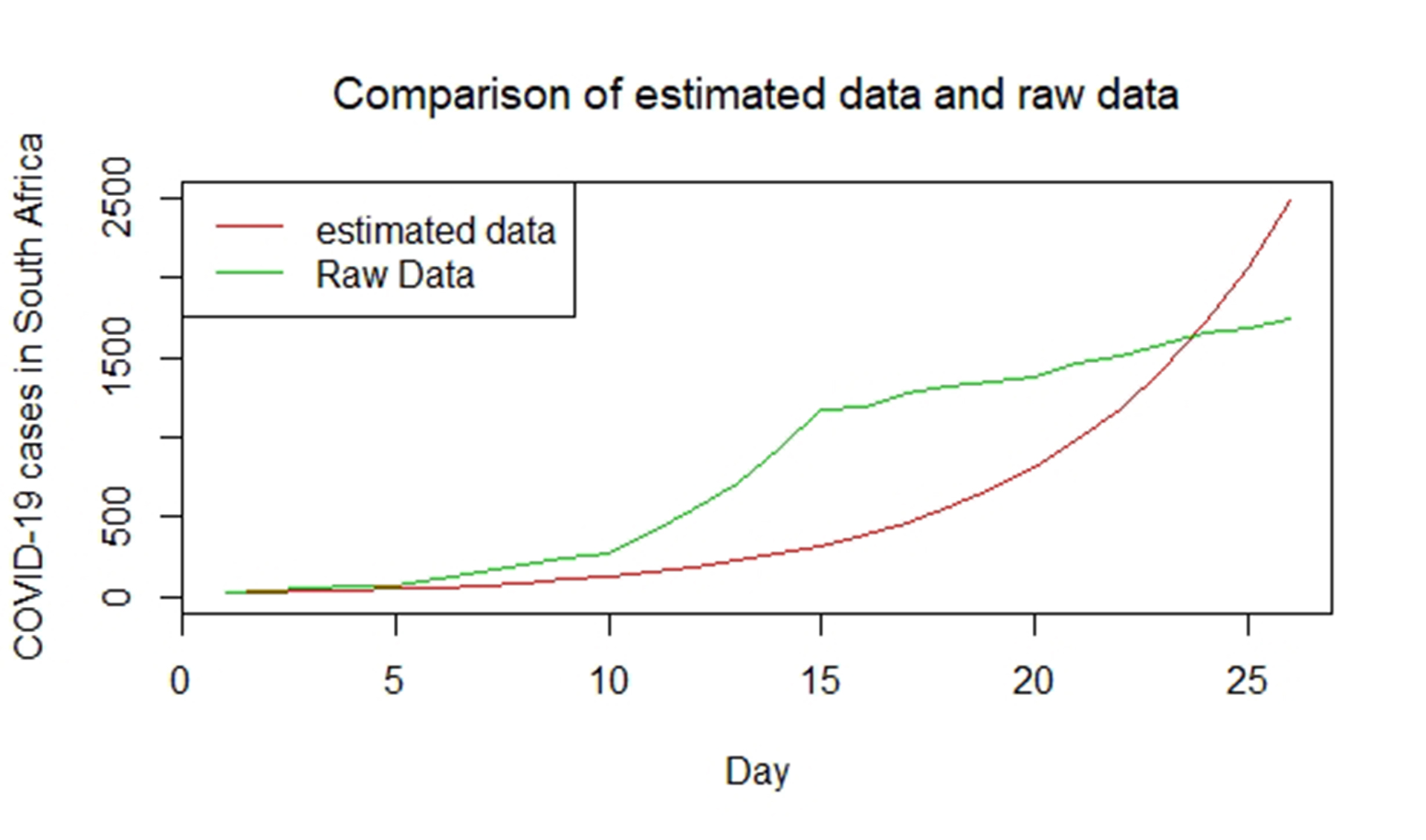

Supplement: Supplementary file 12 [file Image_12.JPEG]

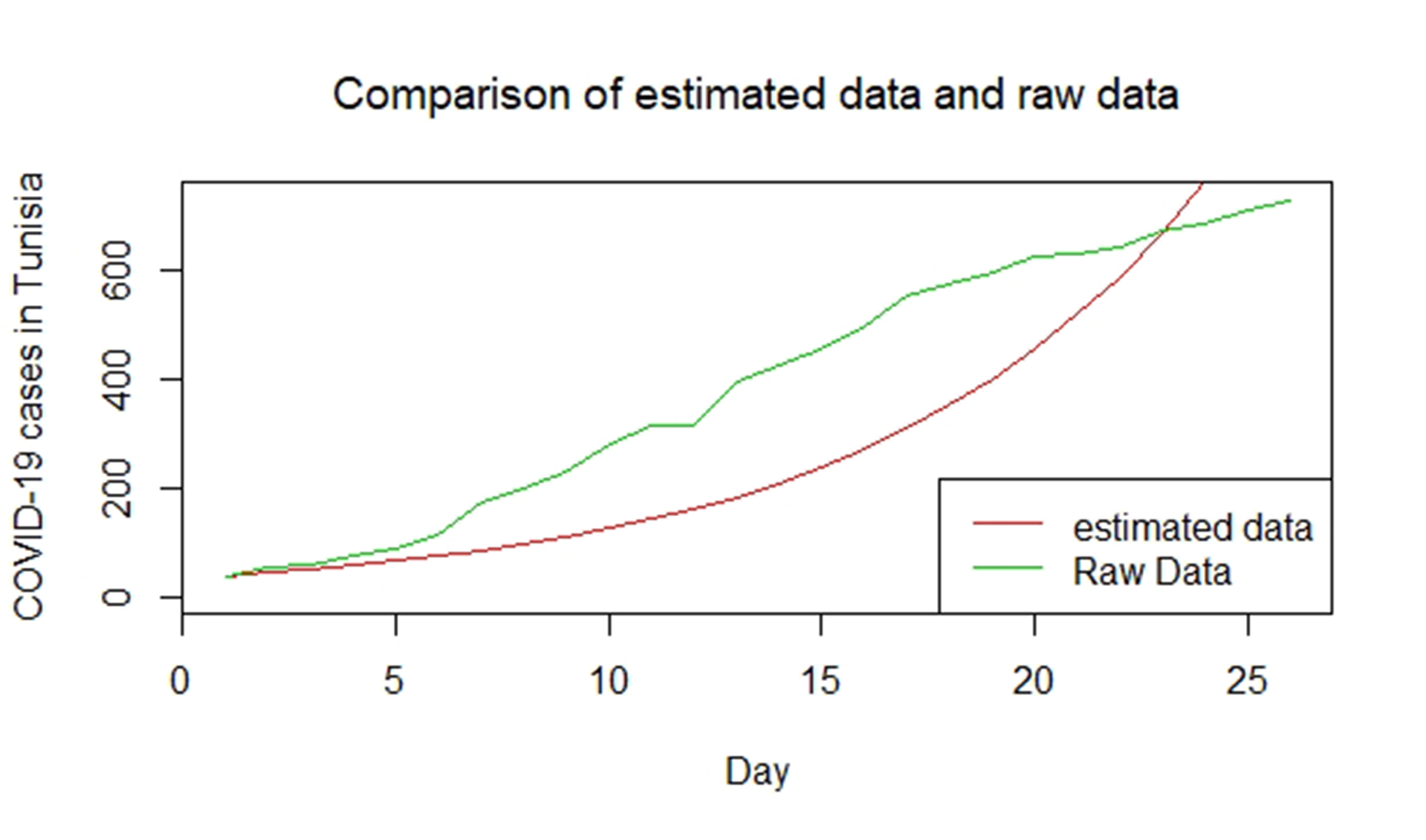

Supplement: Supplementary file 13 [file Image_13.JPEG]
